# The effect of X-linked dosage compensation on complex trait variation

**DOI:** 10.1038/s41467-019-10598-y

**Published:** 2019-07-08

**Authors:** Julia Sidorenko, Irfahan Kassam, Kathryn E. Kemper, Jian Zeng, Luke R. Lloyd-Jones, Grant W. Montgomery, Greg Gibson, Andres Metspalu, Tonu Esko, Jian Yang, Allan F. McRae, Peter M. Visscher

**Affiliations:** 1 Institute for Molecular Bioscience, The University of Queensland, Brisbane, 4072 QLD Australia; 2 School of Biology and Centre for Integrative Genomics, Georgia Institute of Technology, Atlanta, GA 30332 USA; 3 Estonian Genome Centre, Institute of Genomics, University of Tartu, Tartu, 51010 Estonia; 4 Queensland Brain Institute, The University of Queensland, Brisbane, 4072 QLD Australia

**Keywords:** Dosage compensation, Gene expression, Genomics, Heritable quantitative trait

## Abstract

Quantitative genetics theory predicts that X-chromosome dosage compensation (DC) will have a detectable effect on the amount of genetic and therefore phenotypic trait variances at associated loci in males and females. Here, we systematically examine the role of DC in humans in 20 complex traits in a sample of more than 450,000 individuals from the UK Biobank and 1600 gene expression traits from a sample of 2000 individuals as well as across-tissue gene expression from the GTEx resource. We find approximately twice as much X-linked genetic variation across the UK Biobank traits in males (mean *h*^2^_SNP_ = 0.63%) compared to females (mean *h*^2^_SNP_ = 0.30%), confirming the predicted DC effect. Our DC estimates for complex traits and gene expression are consistent with a small proportion of genes escaping X-inactivation in a trait- and tissue-dependent manner. Finally, we highlight examples of biologically relevant X-linked heterogeneity between the sexes that bias DC estimates if unaccounted for.

## Abstract (web summary)

Dosage compensation (DC) on the X chromosome has predictable effects on genetic and phenotypic trait variance. Here, the authors use information for 20 quantitative traits in the UK Biobank and across-tissue gene expression to compare X-linked heritability and the effects of trait-associated SNPs between the sexes.

## Introduction

In eutherian mammals, including humans, females inherit two copies of the X chromosome and males only one. To balance allele dosage differences in X-linked genes between the sexes, dosage compensation (DC) mechanisms have evolved to randomly inactivate one of the X chromosomes in female cells^1,2^. Due to random choice and the stability of the X chromosome inactivation (XCI) in mitotically derived cell lineages, the paternal or maternal alleles are expected to be mono-allelically expressed in different cell populations approximately 50% of the time, making females functionally haploid mosaics with respect to the X-linked genes. Notably, visible examples of diverse patterns of mosaicism in females heterozygous for easily distinguishable, monogenic, X-linked traits, such as X-linked fur colour in mice, were among the first lines of evidence for the hypothesis of random XCI^2^.

The process of XCI is initiated during early embryogenesis and is mediated via expression of the non-coding RNA X inactivation-specific transcript (XIST) from the X inactivation centre of the future inactive X chromosome^3–5^, and a combination of epigenetic modifications including histone modifications and DNA methylation to achieve transcriptional silencing of the X-linked genes^6,7^. The inactive X chromosome can be observed as a tightly condensed heterochromatin body, also known as a Barr body^8^. However, an increasing body of evidence suggests that XCI is incomplete with significant variability across tissues and even between the single cells^9,10^. Studies focused on identifying X-chromosome inactivation profiles^10–14^ agree that only 60–75% of the assessed X-linked genes are subject to complete silencing on the inactive copy of the X chromosome, posing the question of how escape from XCI affects complex trait variation and sex differences.

From the perspective of quantitative genetics, DC at loci affecting a trait has a predictable effect on differences in genetic and therefore phenotypic trait variances in males and females and on the resemblance between male-male, male–female and female-female relatives^15–17^. The difference in ploidy between males and females implies that a segregating mutation with alleles *B* and *b* (with frequency *p* and (1-*p*), respectively) occurs as haplotypes *B-* and *b-* in males and as genotypes *BB*, *Bb* and *bb* in females. If the additive value of allele *B* on a trait is *α* then the haplotype values of *B-* and *b-* are (apart from a constant) *α* and 0, respectively, and the variance due to this mutation in males is *p*(1-*p*)*α*^2^. In females, the genotype values are 2*α*, *α* and 0 for *BB*, *Bb* and *bb*, respectively, with a variance of 2*p*(1-*p*)*α*^2^, twice of that in males. In contrast, due to random XCI in females and the resulting mosaicism, the three genotypes (*BB*, *Bb* and *bb*) have genotypic values of *B*-, ½(*B*-) + ½(*b*-), and *b*-, respectively, so values of *α*, ½*α*, 0, leading to a variance of 2*p*(1-*p*)(½ *α*)^2^ = ½*p*(1-*p*)*α*^2^, half of that in males. Therefore, ploidy and XCI have opposite effects on the variance difference between males and females at trait loci on the X chromosome. For complex traits, where genetic variation is contributed by a large number of variants with small effects, the overall heritability attributable to the X chromosome will depend on the inactivation state of the X-linked loci affecting a trait. The limiting case of DC at all associated loci, which we refer to as full DC (FDC), is therefore predicted to lead to twice as much genetic variation in males compared to females, whereas the complete lack of inactivation, where only the ploidy difference matters, would lead to twice the variation in diploid females compared to haploid males^16^. Moreover, the double dosage of the genes that escape from DC would affect the mean value of the trait of interest in females compared to males and may contribute to sex differences in complex traits.

In this study, we leverage information on 20 complex phenotypes in the UK Biobank (*N* = 208,419 males and *N* = 247,186 females), 1649 gene expression traits in whole-blood (*N* = 1084 males and *N* = 1046 females), and a mean of 808 gene expression traits across 22 tissue-types in GTEx (mean *N* = 142 males and mean *N* = 85 females) to compare the predicted effect of random X-inactivation to the empirical data. The expected ratio of male–female (M/F) heritability attributable to the X chromosome (i.e. the DC ratio, DCR) is equal to 2 in the case of FDC and 0.5 in the case of no DC. We perform a sex-stratified X-chromosome-wide association analysis (XWAS) for all traits to estimate DCR in high-order UK Biobank traits and compare M/F effect estimates of associated SNPs for both phenotypic and gene expression traits. Our results confirm the predicted effect of DC on X-linked trait variation and are consistent with a small proportion of genes partially or fully escaping from X-inactivation.

## Results

### Evidence for DC in complex traits

**Fig. 1:**
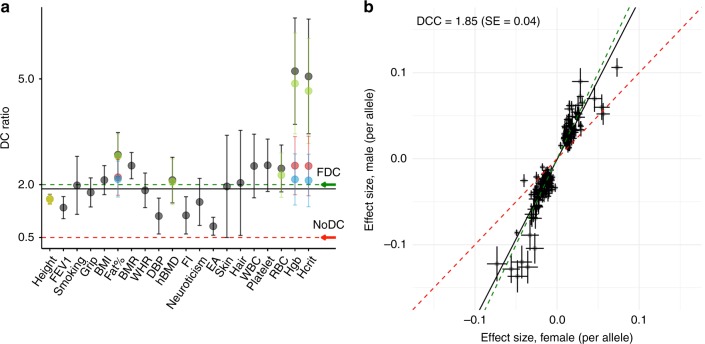
Estimates of dosage compensation (DC) ratio and dosage compensation coefficient (DCC) for the UK Biobank (UKB) traits. **a** DC ratio with 95% confidence intervals (DC ratio +/− 1.96*SE) for 20 UKB traits as estimated using summary statistics from the association analyses. The black points indicate the DC ratio estimated using all SNPs on the X chromosome (non-pseudoautosomal region, non-PAR). For Height, Fat%, hBMD, RBC, Hgb and Hcrit the DC ratios are re-estimated excluding the SNPs in the regions of identified heterogeneity (Supplementary Table 4) and presented as coloured points (green: excluding region 1; yellow: excluding region 2; red: excluding region 3 or 4; blue: excluding region 1 and 3 or 4). The green and red dashed lines indicate the expectations under full DC (FDC) and lack of DC (No DC), respectively. The black solid line is the mean DC ratio (=1.88) after accounting for heterogeneity. **b** Male and female per-allele effect estimates (in standard deviation units) (+/- SE) are compared for the lead SNPs identified in the combined discovery analysis (*M* = 251). The SNPs located in the regions of heterogeneity for the six traits mentioned above are excluded. The green and red dashed lines indicate the expectations under FDC and no DC, respectively. The black line represents DCC (1.85, SE = 0.04). SE, standard error. *M*, number of lead SNPs. Traits: standing height (Height), forced expiratory volume in 1-second (FEV1), smoking status (Smoking), hand grip strength, right (Grip), body mass index (BMI), body fat percentage (Fat%), basal metabolic rate (BMR), waist to hip ratio (WHR), diastolic blood pressure (DBP), heel bone mineral density T-score (hBMD), fluid intelligence score (FI), neuroticism score (Neuroticism), educational attainment (EA), skin colour (Skin), hair colour (Hair), white blood cell (leukocyte) count (WBC), platelet count (Platelet), red blood cell (erythrocyte) count (RBC), haemoglobin concentration (Hgb), Haematocrit percentage (Hcrit)

We first performed a sex-stratified genome-wide association analysis for 20 quantitative traits in the UK Biobank (UKB) **(**for trait information see Supplementary Table 1), and estimated ratios of male to female X-linked SNP-heritabilities (*h*^2^_SNP_) from the summary statistics. Depending on the amount of DC on the X chromosome in females, this ratio is expected to take a value between 0.5 (no DC) and 2 (FDC). We refer to this as the DC ratio (DCR). For 19 out of 20 traits, the DCR estimates on the X chromosome (non-pseudoautosomal region, non-PAR) were significantly different from the expectation for no DC (i.e. DCR = 0.5), and consistent with evidence for DC between sexes on the X chromosome and its detectable effect on phenotypic trait variation (Fig. 1a, shown as the black points). We validated our DCR summary statistics approach by calculating DCR from the sex-specific estimates of *h*^2^_SNP_ derived from GCTA-GREML^18^ on individual-level data from up to 100,000 unrelated individuals (Supplementary Table 2). From this analysis, we found the X-linked genetic variance of the complex traits to be low in general, but detectable in this large sample with the mean *h*^2^_SNP_ attributable to the X chromosome of 0.63% (SD = 0.33%) and 0.30% (SD = 0.20%) across the UKB traits in males and females, respectively. Notably, using height and BMI in males as an example, we established that the observed lower variation contributed by the X chromosome compared to autosomes of similar length is a result of haploidy in males and not of smaller per-allele effects at segregating trait loci (see Supplementary Fig. 1 and Supplementary Note 1). X-chromosome specific *h*^2^_SNP_ estimates were significantly different from zero for all 20 traits in males and for 18 traits in females (the *h*^2^_SNP_ estimates for the skin and hair colour traits did not significantly differ from zero in the female-specific analysis) (Supplementary Table 2). For these 18 traits, we found a strong overall correlation between DCR estimates obtained with the two methods (Pearson correlation *r* = 0.78, Supplementary Fig. 2).

From the analysis based on summary statistics, the mean DCR for the X chromosome across 20 traits was 2.23 (SD = 1.13), consistent with the predicted effect of DC. In contrast, the estimates of the ratios for autosomal SNP-heritability varied from 0.66 to 1.17 with mean of 0.95, in agreement with a limited difference in *h*^2^_SNP_ between the sexes in autosomal loci (Supplementary Table 3). We observed DCR on the X chromosome significantly different from expected values under both hypotheses (full and no DC) for nine traits (Fig. 1a, black points). While for standing height (height), forced expiratory volume in 1 second (FEV1), DBP, FI and EA the DCR estimates ranged between 0.5 and 2, indicating partial DC, the values larger than 2 (body fat percentage (Fat%), basal metabolic rate (BMR), haemoglobin concentration (Hgb) and haematocrit percentage (Hcrit)) could not be explained under either of the DC models. We therefore sought an alternative explanation for these observations.

When estimating the DCR, we assumed that the genetic correlation (*r*_g_) between males and females is equal to one, and that any difference in the genetic variance is due to differences in dosage (i.e. number of active copies) of the X-linked genes. We estimated autosomal (*r*_gA_) and X-linked (*r*_gX_) genetic correlations in our sample using the GWAS summary statistics (see Methods). The evidence for autosomal genetic heterogeneity in complex trait is limited^19,20^ and our estimates of *r*_gA_ between sexes are similar to published results (mean *r*_gA_ = 0.92, SD = 0.06 across 20 traits, Supplementary Table 3). However, we found lower genetic correlation across the 20 traits on the X chromosome (*r*_gX_ = 0.81, SD = 0.14) (Supplementary Table 3). The smallest *r*_gX_ estimates correspond to Hcrit (*r*_gX_ = 0.51, SE = 0.05), Fat% (*r*_gX_ = 0.57, SE = 0.05), red blood cell count (RBC) (*r*_gX_ = 0.64, SE = 0.07) and Hgb (*r*_gX_ = 0.65, SE = 0.04). These relatively low *r*_gX_ estimates may indicate local differences in genetic variance between males and females on the X chromosome that is independent of DC, which may explain the observed extreme DCR estimates for these traits. We thus explored biological heterogeneity as an explanation for these observations.

### Biological heterogeneity on the X chromosome

**Fig. 2:**
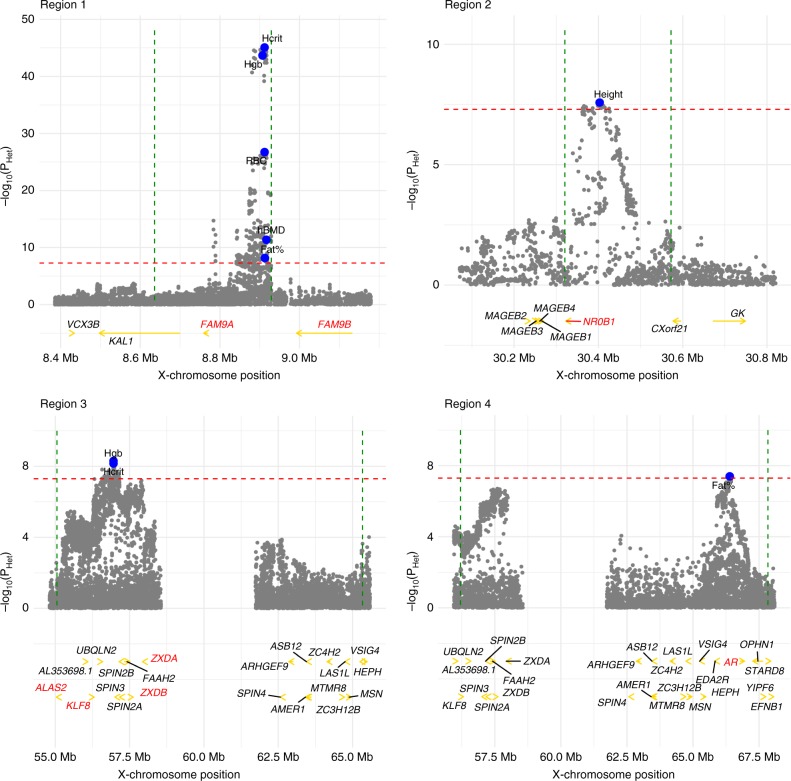
Four regions of heterogeneity (+/−250 kb) on the X chromosome. For each trait, regions of heterogeneity were identified as all SNPs within a region of linkage disequilibrium (LD) *R*^2^ > 0.05 to the SNP with highest evidence of significant heterogeneity (Supplementary Table 4). In each region the heterogeneity *P*-values (*P*_Het_) are plotted (grey dots) for all traits with significant heterogeneity in that region. The top SNPs for each trait are shown in blue. The genes discussed in the Supplementary Note 2 are highlighted in red. In region 3, only the *ALAS2* gene and genes with X-chromosome position > 56 Mb are shown for simplicity (the omitted 15 genes are: *ITIH6*, *MAGED2*, *TRO, PFKFB1*, *APEX2*, *PAGE2B*, *PAGE2*, *FAM104B*, *MTRNR2L10*, *PAGE5*, *PAGE3*, *MAGEH1*, *USP51*, *FOXR2*, *RRAGB*). The red dashed line represents the significance threshold (*P*_Het_ = 5.0 × 10^–8^). The green dashed lines represent the boundaries of the regions. Traits: Haematocrit percentage (Hcrit), haemoglobin concentration (Hgb), red blood cell (erythrocyte) count (RBC), heel bone mineral density T-score (hBMD), body fat percentage (Fat%), standing height (Height)

To investigate sex-specific genetic architectures on the X chromosome, we tested for heterogeneity in male and female SNP effects under the null hypothesis of no difference. A total of 6 traits (Hcrit, Fat%, RBC, Hgb, height and heel bone mineral density T-score (hBMD)) showed evidence for heterogeneity. We identified four regions of heterogeneity, two of which overlap due to the complex linkage disequilibrium (LD) structure in the centromere region (Fig. 2, Supplementary Table 4).

Sex-related differences between males and females are most likely to arise due to naturally differing sex hormone levels, thus we examined the evidence for hormonal regulation in these regions (Supplementary Note 2). Overall, at least three of the four regions of detected heterogeneity on the X chromosome show evidence of male-specific and/or androgen-related effects on the traits, and thus may not reflect an effect of DC, but rather biological differences between the sexes which are mediated by sex hormones. We therefore re-estimated DCR for Hcrit, Fat%, RBC, Hgb, height and hBMD after excluding these regions of heterogeneity (Supplementary Table 5, Fig. 1a). While there was no significant change in DCR for height, we found a significant decrease in DCR and an increase in genetic correlation for the remaining five traits. After re-estimating DCR for the 6 traits, our mean estimate of DCR across all 20 UK Biobank traits decreased from 2.23 (SD = 1.13) to 1.88 (SD = 0.49). These observations are consistent with the hypothesis that a disproportionate amount of male-specific genetic variance in these regions is at least partially hormonally influenced.

### DC at complex trait-associated loci

In addition to testing for differences in overall X-linked variance between the sexes, we can estimate a DC parameter *d* such that *β*_m_ = *dβ*_f_ (see Supplementary Methods) for genome-wide significant trait-associated SNPs. We did this by regressing the male-specific effect estimates onto the effects of the same markers estimated in female-specific analysis, weighted by the inverse of the variance of male-specific effect estimates. We define this regression slope as the dosage compensation coefficient (DCC), which is expected to take on values between 1 (escape from XCI) and 2 (FDC).

We applied conditional and joint association analysis (GCTA-COJO)^21^ to the summary statistics from the male-, female- and combined male–female discovery analysis to select jointly significant trait-associated SNPs (hereafter called lead SNPs) for each of the 20 UKB traits. This identified 153 (male discovery) and 61 (female discovery) lead SNPs on the non-PAR X chromosome at a genome-wide significance (GWS) level of *P*_COJO_ < 5.0 × 10^–8^ across the tested phenotypic traits (Supplementary Table 6, Supplementary data 1–2). That is, more than twice the number of non-PAR lead SNPs were identified in males compared to females, indicating that a larger proportion of per-locus and therefore total genetic variance is explained in males compared to females. In contrast, in the pseudoautosomal region (PAR), we identified two lead loci in males, while eight of them were detected in female discovery analysis (Supplementary Table 6, Supplementary data 1–2). In the combined male–female discovery analysis, 261 non-PAR and 16 PAR SNPs satisfied our GWS threshold in the GCTA-COJO analysis (Supplementary Table 6, Supplementary data 3). The increased number of lead SNPs in comparison to the sex-stratified analysis indicates concordance of effects from sex-specific analyses. The comparison of sex-specific genetic variance on the X chromosome, highlighting the lead SNPs in the combined set, is illustrated in Supplementary Fig. 3.

We estimated DCC to be 2.13 (SE = 0.08) and 1.47 (SE = 0.07) in the male and female non-PAR discovery analyses, respectively, using the lead SNPs across the analysed complex traits (Supplementary Fig. 4**)**. DCC for the markers identified in the combined male–female analysis was 1.85 (SE = 0.04), consistent with a small proportion of genes variably escaping from X-inactivation among the studied traits (Fig. 1b). For the PAR, although the number of significant associations was small, the sex-specific effects size estimates were similar (Supplementary Fig. 5), consistent with theoretical expectations.

The ratio of the M/F per-allele effect sizes for individual SNPs, which approximates the DC parameter, indicated the evidence for escape from XCI only for a few candidate variants. For instance, SNP rs113303918 in the intron of the *FHL1* gene is significantly associated with WHR in female and the combined male–female analyses (*P*_XWAS, female_ = 6.6x10^–12^ and *P*_XWAS, combined_ = 9.8x10^–14^, respectively), while being only nominally significant in the male-specific analysis (*P*_XWAS, male_ = 4.5 × 10^–5^) and the per-allele effect sizes on WHR are similar in both sexes (effect size ratio = 0.93, SE = 0.26). Similarly, the effect size ratio of SNP rs35318931 (*P*_XWAS, female_ = 2.7 × 10^–17^, *P*_XWAS, male_ = 6.7 × 10^–4^, *P*_XWAS, combined_ = 2.8 × 10^–15^), a possible missense variant in the *SRPX* gene, is 0.63 (SE = 0.20) consistent with escape from XCI for WHR. Assuming that these SNPs are the causal variants, the observed effect size estimates may indicate potential escape from XCI for *FHL1* and *SRPX*. Interestingly, for height (effect size ratio = 2.12, SE = 0.35; *P*_XWAS, combined_ = 1.9 × 10^–37^) and BMR (effect size ratio = 3.26, SE = 1.21; *P*_XWAS, combined_ = 6.6 × 10^–12^) the results for the SNP rs35318931 in the *SRPX* gene were indicative of DC. Consistent with these observations*, SRPX* is annotated with Variable XCI status in^10,13^. For *FHL1*, although, annotated as Inactive in^10^, findings from two earlier studies^13,14^ show that XCI is incomplete. Moreover, heterogeneous XCI of *FHL1* is detected in single cells and across tissues^10^.

Previously, a locus near the *ITM2A* gene (SNP rs1751138, bp 78,657,806) was proposed as a potential XCI-escaping locus associated with height^22^. In our sex-stratified and combined analyses with a sample size an order of magnitude larger, the lead marker for height was a nearby SNP rs1736534 located approximately 100 bp upstream of the previously reported SNP rs1751138. The estimated M/F effect size ratio for the both variants was 1.75 (SE = 0.11) (*β*_height, male_ = −0.086, SE = 0.004 and *β*_height, female_ = −0.049, SE = 0.002), providing evidence against extensive escape of *ITM2A* from XCI.

About one-third of the identified lead SNPs were physically located within X-linked gene regions. For these SNPs, we assigned the XCI status according to the reported XCI status of the corresponding genes^10^ and compared the effect size ratios between Escape/Variable and Inactive genes. The results remained similar between the two groups of genes (Supplementary Fig. 6). A notable disadvantage of this approach is that the physical location of a SNP within a gene region does not necessarily indicate a causal variant for a complex trait. In contrast, an expression quantitative loci (eQTL) analysis avoids this, as there is no ambiguity between mapped SNPs and genes, and thus the annotation of XCI status.

### Evidence for DC in gene expression

**Fig. 3:**
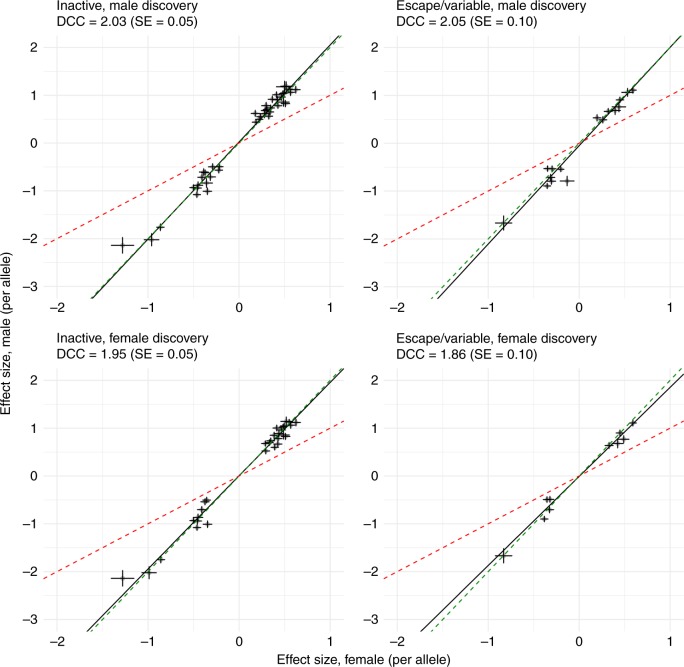
Dosage compensation coefficient (DCC) for eQTLs from blood samples. Comparison of per-allele effects from sex-specific analyses (+/- SE) for *cis*-eQTLs identified in CAGE whole blood. A total of 57/74 and 40/51 eQTLs (*P*_eQTL_ < 1.6 × 10^–10^) in males and females, respectively, had either Escape, Variable, or Inactive status using annotations from^10^. For 41 inactive eQTLs in the male discovery, DCC is 2.03 (SE = 0.05), and for 16 escape or variable escape eQTLs, DCC is 2.05 (SE = 0.10). For 30 inactive eQTLs in the female discovery, DCC is 1.95 (SE = 0.05), and for 10 escape or variable escape eQTLs, DCC is 1.86 (SE = 0.10). The red dashed line represents the expectation under the escape from X chromosome inactivation (XCI) model. The green dashed line represents the expectation under full dosage compensation model. The black line is the regression line. SE, standard error

We extended our DCC analysis to gene expression traits and performed a sex-stratified *cis*-eQTL analysis for 1639 X-chromosome gene expression probes (28 of them in PAR) measured in whole blood from the Consortium for the Architecture of Gene Expression (CAGE)^23^. For each gene expression probe, we identified the top associated X-chromosome SNP with MAF > 0.01 that satisfied the Bonferroni significance threshold of *P*_eQTL_ < 1.6 × 10^–10^ (i.e., 0.05/(1639 × 190,245)) in the discovery sex (hereafter called eQTL), and extracted the same eQTL in the other sex and calculated DCC for M/F eQTL effect size estimates. We observed DCC of 1.95 (SE = 0.04) for 51 eQTLs (48 unique SNPs) in the female discovery analysis, and DCC of 2.07 (SE = 0.04) for 74 eQTLs (68 unique SNPs) in the male discovery analysis (Supplementary Fig. 7), consistent with expectations from FDC and in agreement with our observations in high-order complex traits. We did not identify eQTLs for probes in PAR. Partitioning the non-PAR eQTLs based on reported XCI status of the corresponding genes^10^ did not alter our results (Fig. 3). In particular, for eQTLs annotated to escape XCI, DCC estimates were approximately two, consistent with FDC. Interestingly, for 6 eQTLs identified in the male discovery analysis and annotated to escape XCI (*USP9X*, *EIF2S3*, *CA5B*, *TRAPPC2*, *AP1S2*, and *OFD1*), we observed higher expression in females compared to males (*P*_sex_diff_ < 3.1 × 10^–3^, i.e., 0.05/16), as expected for genes that escape from XCI. However, we found significant differences between the eQTL effect estimates of the top associated SNP on gene expression after correction for mean differences in expression between the sexes (genotype-by-sex interaction *P*_GxS_ < 3.1 × 10^–3^), which is consistent with DC. This suggests that sex differences in the expression of these genes may not be due to escape from XCI (Supplementary Fig. 8). Finally, DCC estimates remained consistent when restricting to genes that show either significant (*P*_sex_diff_ < 10^–3^) female-bias (male discovery analysis: 21 eQTLs with DCC of 1.98 (SE = 0.10); female discovery analysis: 16 eQTLs with DCC of 1.86 (SE = 0.09)) or no mean difference (*P*_sex_diff_ > 0.05) in expression between the sexes (male discovery analysis: 31 eQTLs with DCC of 2.14 (SE = 0.07); female discovery analysis: 29 eQTLs with DCC of 2.03 (SE = 0.06)). Full details of the eQTLs in whole blood can be found in Supplementary data 4 and 5.

**Fig. 4:**
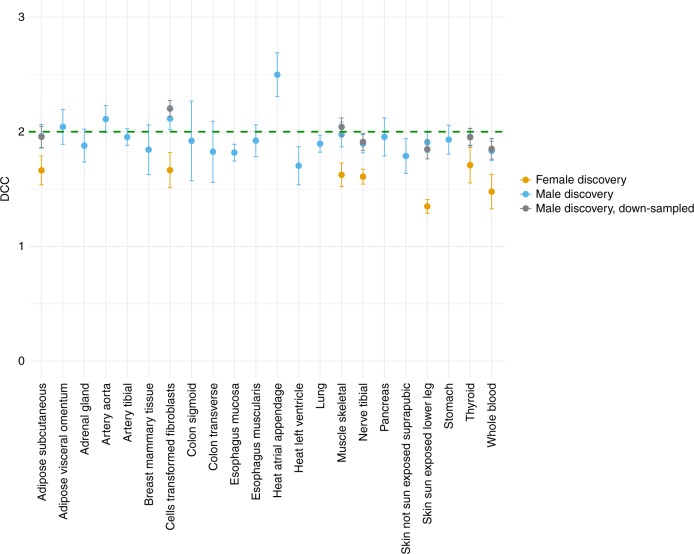
Dosage compensation coefficient (DCC) for eQTLs across tissues. DCC is estimated for tissues with at least three eQTLs that satisfied the within tissue Bonferroni significance threshold in each of the 22 tissue-types. A mean of 28 (SD = 18) eQTL are identified in the male discovery analysis giving a mean DCC of 1.94 (SD = 0.16) across 22 tissues. A mean of 5 (SD = 0.82) eQTLs are identified in the female discovery analysis giving mean DCC of 1.59 (SD = 0.13) across seven tissues. The coloured bars represent the standard errors. Males were down-sampled 100 times so that the proportions match that of females within each of the seven tissues, and mean DCC is calculated across the 100 replicates. The grey bars represent the standard deviation across 100 replicates. SD, standard deviation

We validated our results in 22 tissue samples from GTEx (v6p release) for which within tissue sample size was greater than *N* = 50 in both males and females (Supplementary data 6). We estimated DCC for tissues with at least three eQTLs that satisfied the within tissue Bonferroni significance threshold in the discovery sex in each of the 22 tissue-types. No eQTLs were identified for transcripts in PAR. A mean of 28 (SD = 18) eQTLs were identified in the male discovery analysis across the 22 tissues. We observed a mean DCC of 1.94 (SD = 0.16) across 22 tissues in the male discovery analysis, with the 95 percent confidence intervals for 20 tissues overlapping 2 (Fig. 4). Heart (atrial appendage) tissue was an outlier, with DCC of 2.50 (SE = 0.19). In contrast, a mean of 5 (SD = 0.82) eQTLs were identified in females across the seven tissues. A mean DCC of 1.59 (SD = 0.13) across the seven tissues was observed in the female discovery analysis, with only the 95 percent confidence interval for thyroid tissue overlapping 2. We verified that the difference in estimated DCCs is not due to differences in sample size between the sexes by down-sampling males so that the proportions match that of females within each of the seven tissues and calculating mean DCC across 100 replicates (Fig. 4). We did not observe enrichment for Escape/Variable eQTLs identified in the male or female discovery analyses by hypergeometric test (Supplementary data 7). These results were consistent when the top eQTLs were chosen among all tissues in the discovery sex and compared to the same eQTL from the same tissue in the other sex (Supplementary Fig. 9). Finally, a combined male–female analysis using a 2 degree-of-freedom eQTL interaction model identified a mean of 41 eQTLs (SD = 20), which gave a mean DCC of 1.75 (SD = 0.14) across the 22 tissues. A total of six tissues had the 95 percent confidence interval overlapping 2 (Supplementary Fig. 10). These results are indicative of partial escape from X-inactivation. Full details of the eQTLs across tissues can be found in Supplementary data 6–8.

Finally, we compared our results to those from a sex-stratified autosomal *cis*-eQTL analysis in 36,267 autosomal gene expression probes in CAGE whole blood. A similar number of eQTLs with *P*_eQTL_ < 10^–10^ were identified in both sexes (3116 in the male discovery vs. 3165 in the female discovery), indicating that an approximately equal proportion of autosomal genetic variance per locus is explained in each of the sexes. As expected, DCC in the male and female discovery was 1.00 (SE = 2.3x10^–3^) and 0.94 (SE = 2.3 × 10^–3^), respectively, indicating that the autosomal eQTL effect sizes are approximately equal in males and females (Supplementary Fig. 11).

### Summary-data based mendelian randomisation

As noted above, there may be some ambiguity in mapping the associated variants to the genes based on its physical location, since the true causal variants may be masked by the local LD-structure or may exert the regulatory action on both near and distantly located genes^24,25^. To investigate this, we aimed to integrate the GWAS data from the complex trait analysis and the eQTL data from the CAGE whole-blood analysis, to prioritize genes whose expression levels are associated with complex traits due to pleiotropy. This would then allow for the XCI status to be assigned to the relevant putative causal gene. The summary data-based Mendelian randomisation (SMR) analysis^24^ identified 18 genes (tagged by 20 probes) that are significantly (*P*_SMR_ < 3.0 × 10^–5^ (0.05/1639) and *P*_HEIDI_ > 0.05) associated with 13 complex traits (total of 36 associations) in the combined male–female sample (Supplementary data 9). For males, associations between 13 genes (15 probes) and 11 traits satisfied our significance thresholds (total of 23 associations) (Supplementary data 10), while the female analysis identified 4 significant pleiotropic associations between 3 genes (3 probes) and 4 traits (Supplementary data 11). The effect estimates for the pleiotropic SNPs identified in SMR analyses are illustrated in Supplementary Fig. 12. The estimated DCC for these variants is similar to the results estimated with all jointly significant SNPs from the GCTA-COJO^21^ analysis (Fig. 1b, Supplementary Fig. 4).

Our SMR analysis linked many SNPs located in the intergenic regions to the expression of specific genes, however, a number of SNPs physically located within a gene were found to be associated with expression of another gene (e.g. a SNP in *TMEM255A* was an eQTL for *ZBTB33* whose expression is associated with traits skin and hair colour). This also included previous signals in escape genes being assigned to inactive genes (e.g. the SNPs physically located in the annotated escape gene, *SMC1A*, was associated with the expression of the inactive *HSD17B10* for BMI, BMR, Fat% and EA in the combined male–female SMR analysis). The expression of only two genes (*MAGEE1* and *PRKX*) annotated as Variable or Escape from XCI, respectively, showed evidence for pleiotropic association with a phenotypic trait (hand grip strength (Grip) and white blood cells (WBC), respectively) due to a shared genetic determinant (*MAGEE1*: *P*_SMR,combined_ = 2.1 × 10^–6^, *PRKX: P*_SMR,combined_ = 8.7x10^–6^, Supplementary Fig. 12, Supplementary data 9). The estimated effect size ratio (2.84, SE = 0.85) for the variant rs757314 (mediated by *MAGEE1* expression levels) on hand grip strength was not consistent with the escape from X-inactivation (the expected ratio for an escape gene is 1). For the SNP rs6641619 (associated with *PRKX* expression and WBC), the effect size ratio estimate was 1.33 (SE = 0.44), which is indicative of partial escape from X-inactivation.

Variants near *ITM2A* were shown to be associated with height in a previous study^22^ and with height, BMR, Grip, WHR and FEV1 in the current XWAS analysis. The results of the combined SMR analysis for the *ITM2A* gene-trait association further supported the evidence for *ITM2A* inactivation (Supplementary Note 3, Supplementary data 12–13).

## Discussion

The theoretically predicted effect of random X-inactivation in female cells is a two-fold reduced amount of additive genetic variance in females compared to males, whereas escape from XCI would increase genetic variance in females. For example, an escape rate of 15% is expected to reduce the DCR to 1.78 (=2*0.85 + ½*0.15) and the mean effect size ratio at the trait-associated loci to 1.85 (=2*0.85 + 1*0.15). Having analysed phenotypes with varying degree of polygenicity, we confirmed the predicted effect of DC on X-linked trait variation both in moderately and highly polygenic traits (gene expression and phenotypic traits in the UKB, respectively). The two strategies that we use to estimate DC are the overall ratio of M/F X-linked heritabilities (i.e. the DC ratio) and the comparison of the individual effects for trait-associated variants between the sexes (i.e. the effect size ratio and DCC). These are parameterisations of the same effect, the former based upon the variance contributed by all X-linked trait loci and the latter based upon per-allele effect sizes of trait-associated loci. Previous studies have demonstrated that ~1% of the phenotypic variance for traits such as height and BMI, is attributable to the X chromosome^22,26^. However, attempts to disentangle the relationships of additive genetic variance between the sexes in high-order complex traits were limited in power due to moderate sample sizes and/or computational challenges^22,26^. Here, a large the sample of >205,000 males and >245,000 females allowed us to identify a statistically significant contribution of the X chromosome to the total trait heritability and to make further inferences on the DCR in complex traits. While we observed good overall evidence for DC across the complex traits, a number of outliers were present in our analysis. First, we observed unexpectedly high ratios of male to female genetic variance for some of the traits. This is likely to be attributable to the male-specific genetic control for some X-linked regions and thus is not informative on DC. Additionally, while the region comprising a testosterone-associated locus (near *FAM9A/FAM9B* genes) had the strongest evidence of heterogeneity, its removal had modest effect on DCR, while the exclusion of the genomic region near the centromere had the strongest effect. In addition to possible androgen-specific influence of this region, the tight LD structure may contribute disproportionately to sex-specific genetic variance. Second, we observe DCR supporting possible escape from XCI rather than FDC in brain related traits, such as educational attainment (EA) and fluid intelligence (FI), and also diastolic blood pressure (DBP). Consistently, brain tissues have the highest X chromosome to autosome expression ratio, followed by heart^27,28^, in agreement with an enhanced X-chromosome role in cognitive functions. This indicates that the targets of segregating causal variants may be trait- and tissue-dependent. Overall, if the causal variants are random with respect to XCI then our results are not consistent with large proportion (e.g. 30%) of genes partially or fully escaping XCI for most of the traits. Alternatively, the genes targets of the associated SNPs may be enriched in non-escaping genes for some traits. Distinguishing between these two competing hypotheses would require knowledge of the underlying causal variants, thus limiting our interpretation of the results.

We also found consistent evidence for DC when examining individual trait-associated markers. Interestingly, our results for height associated loci near *ITM2A*, a gene known to be involved in cartilage development, differ from reported evidence for lack of DC^22^ and only a few loci associated with WHR were candidates to be putative escapees. It should be noted, however, that the genetic correlation for WHR on both autosomes and the X chromosome is markedly low, which may reflect the sex-specific genetic control for this trait. However, unless we establish the underlying causal variants, we are not able to reliably infer the inactivation state of a single gene, since trait-associated loci can act over long distances^25,29^, and *cis*-acting variants only tag causal variants through LD. Moreover, it is still unclear if the XCI status of a SNP is most relevant to the causal variant or its gene target, which may differ with respect to XCI status.

In contrast to the high-order complex traits, gene expression traits have a notably different genetic architecture with as much as 65% of the expression variance for a gene explained by a single SNP, thus potentially violating the (polygenic) modelling assumptions for a DCR analysis, and thus was not included as part of this study. We were, however, able to leverage information from eQTLs to show that DCC estimates in gene expression are consistent with expectations from DC, and in agreement with our observations in high-order complex traits and previous eQTL studies^30,31^. These results were broadly consistent across multiple tissue-types, where a larger number of eQTLs were identified in males compared to females and, in the male discovery analysis, DCC is ~2.

Across both the high-order complex traits and gene expression traits, we observed DCC estimates larger than 2 in the male discovery analyses and smaller than 2 in the female discovery analyses. This may be attributed to a combination of partial escape from XCI and “winner's curse” of the XWAS analysis. For example, any loci that partially escape XCI in females would be preferentially selected in the female discovery analysis due to increased statistical power of detection, and thus bias DCC estimates towards 1. Further, winner’s curse would upwardly bias the per-allele effect estimates in the discovery sex compared to the corresponding estimates in the other sex, which may also affect DCC estimates. The combined male–female analyses attempts to avoid the caveats of the sex-stratified analyses and provides unbiased estimates of DC as the effects are assumed to be present (although with a different effect size) in both males and females.

While identification of new associations between X-linked SNPs and complex traits was not the primary aim of this study, our results show these are readily found and that they cumulatively contribute to trait variation. For example, we find pleiotropic association between expression levels of the *HSD17B10* gene with obesity-related traits (Fat% and BMI) and EA.  Notably, *HSD17B10* encodes a mitochondrial enzyme involved in oxidation of neuroactive steroids, fatty acids as well as sex hormones and its deficiency is implicated in neurodegenerative disorders^32^. Consistently, similar putative causal relationships were recently identified for the autosomal gene *HSD17B12*, where its increased expression of this gene was associated with decreased BMI across 22 tissues^33^. Therefore, comprehensive surveys of sex-stratified X chromosome wide association studies for disease and other traits are likely to be rewarding, and may provide insight into new biology and sex differences. Moreover, since our method for estimating the amount of DC only requires summary statistics from association analyses, the availability of sex-stratified results from XWAS studies can further be informative on the effect and dosage of X-linked variation across a range of complex traits.

## Methods

### Genotype coding

The summary statistics reported in this study were generated with a combination of BOLT-LMM v2.3^34^, GCTA 1.94^18^, and PLINK 1.90^35^, all of which have default settings for the treatment of X-chromosome SNPs. For analyses performed using PLINK, we used the default parameters which codes males as {0,1}, and thus gives the appropriate per-allele effect estimates. For BOLT-LMM and GCTA, the male genotypes were analysed as diploid using a {0,2} coding. This distinction makes no impact on the strength of association (i.e. *P*-values), however, we multiply the effect estimates and the corresponding standard errors from the diploid male-specific analysis by 2, allowing us to report our results as per-allele effect estimates. In all cases, females were coded as {0,1,2}.

### UK Biobank data

Sex-stratified association analyses of 20 complex was performed using the phenotype data on *N*_m_ = 208,419 males and *N*_f_ = 247,186 females of European-ancestry and UKB Version 3 release of imputed genotype data (6871 SNPs in pseudoautosomal region (PAR) and 253,842 SNPs in non-pseudoautosomal region (non-PAR) satisfied our quality control criteria and had minor allele frequency, MAF > 0.01). The phenotypes were adjusted for appropriate covariates and converted to sex-specific *Z*-scores prior to analysis (See Supplementary Table 1 and Supplementary Methods for full details). We used medication data (Data Field: 20003, Treatment/medication code) to exclude individuals taking medications with blood pressure lowering effect from the analysis of DBP. Details regarding classification of UKB medication by WHO Anatomical Therapeutic Classification System were described in ref. ^36^. We extracted those medication classified in C02 (Antihypertensives), C03 (Diuretics), C07 (Beta Blockers), C08 (Calcium channel blockers) and C09 (Agents acting on renin-angiotensin system) category.

### CAGE gene expression data

Gene expression and X-chromosome genotype data were available in a subset of *N* = 2130 individuals of verified European ancestry (*N*_m_ = 1084 males, *N*_f_ = 1046 females) from the CAGE^23^. A total of 36,267 autosomal and 1639 X-chromosome gene expression probes (28 in the PAR) in whole blood were available for analysis following quality control. Gene expression levels were adjusted for PEER factors^37,38^ that were not associated with sex (*P*_sex_diff_ > 0.05) in order to preserve the effect of sex on expression and where available, measured covariates such as age, cell counts, and batch effects. A total of 1,066,905 autosomal HapMap3 SNPs imputed to 1000 Genomes Phase 1 Version 3 reference panel^39^ and 190,245 non-PAR X-chromosome SNPs (MAF > 0.01) imputed to the Haplotype Reference Consortium (HRC, release 1.1)^40^ were available for analysis.

### GTEx gene expression data

We used the fully-processed, normalised and filtered RNA-seq data from the Genotype Tissue Expression project (GTEx v6p release). X-chromosome imputed SNP data was obtained from dbGap (Accession phs000424.v6.p1). We restricted our analyses to 22 tissue samples for which within tissue sample size was greater than *N* = 50 in both males and females (Supplementary data 6). A total of 1121 transcripts (31 in the PAR) were expressed in at least one tissue, with a mean of 808 transcripts expressed across all 22 tissues (Supplementary data 6) and a total of 127,808 imputed SNPs in the non-PAR of the X chromosome (MAF > 0.05).

### Sex-stratified XWAS

Summary statistics were generated for 20 complex traits in the UK Biobank using BOLT-LMM v2.3^34^ for the X-chromosome SNPs with MAF > 0.01 in both sexes and using 561,572 HapMap3 SNPs (autosomal and X-chromosomal, pairwise *R*^2^ < 0.9) as model SNPs to estimate genetic relationship matrix (GRM) and correct for confounding.

### Analyses in the combined male–female samples

For complex traits, the results from the sex-stratified association testing were meta-analysed using the inverse-variance weighted method to obtain combined results (performed in R). For combined analysis of gene expression traits, individual data from males and female were pooled together. We assumed FDC for all loci for these analyses.

### Significant SNP-trait associations

GCTA-COJO^21^ was used to identify sets of jointly significant SNPs associated with a trait at GWS threshold *P*_COJO_ < 5.0 × 10^–8^. We use genotypes of a random sample of 100,000 unrelated UKB females of European ancestry as a LD reference and increased the distance of assumed complete linkage equilibrium between markers (window size) to 50 Mb due to higher levels of LD on the X chromosome.

### Estimation of DC ratio from summary statistics

Following^41^, we calculated the DC ratio for 20 complex traits from the summary statistics of the sex-stratified X-chromosome analysis using the following equation:

1 $$\hat \gamma = \frac{{h_m^2}}{{h_f^2}} = \frac{{\left( {\hat \chi _m^2 - 1} \right)N_f}}{{\left( {\hat \chi _f^2 - 1} \right)N_m}}$$

where $\hat \gamma$ is the estimate of the DCR; *h*^2^_*m*_ and *h*^2^_*f*_ are the M/F SNP-heritabilities, respectively; $\hat \chi ^2_m$ and $\hat \chi ^2_f$ are the mean chi-square estimates from the sex-specific association analyses; and *N*_*m*_ and *N*_*f*_ are the corresponding sample sizes in males and females, respectively.

The corresponding standard error is estimated as:

2 $${\mathrm{SE}}\left( {\mathrm{DCR}} \right) = \sqrt {\hat \gamma ^2\left( {\frac{{\mathrm{var}}({\hat {\chi}} _m^2)}{{(\hat \chi _m^2 - 1)^2}} + \frac{{\mathrm{var}}(\hat \chi _f^2)}{{(\hat \chi _f^2 - 1)^2}}} \right)}$$

where the ${\mathrm{var}}(\hat \chi ^2)$ is the variance of the mean test statistic across the X chromosome, which is approximately equal to (2/*M*_eff_)[1 + 2($\hat \chi ^2$− 1)]. *M*_eff_ is the effective number of SNPs, which for the X chromosome is approximately equal to 1300^41^. The DC ratio of 2 indicates the evidence for FDC, while the value of 0.5 implies complete escape from inactivation (no DC).

### Estimation of genetic correlation from summary statistics

We also we obtained an estimator for the male–female genetic correlation on the X chromosome (non-PAR region) or autosomes using the following equation,

3 $$\hat r_g = \frac{{\hat \chi _{mf}^2}}{{\sqrt {(\hat \chi _m^2 - 1)(\hat \chi _f^2 - 1)} }}$$

where, as before, $\hat \chi _m^2$ and $\hat \chi _f^2$ are the mean chi-square estimates from the sex-specific association analyses and $\hat \chi _{mf}^2$ is the cross-product of the Z-statistics from the male and female analyses.

We calculate standard errors using a block jackknife method. We assigned SNPs across the X chromosome to blocks (*B* = 1000) and for each block *k* we calculate an estimate of the genetic correlation $\hat r_g^{(k)}$ as above excluding the SNPs in this block. The standard error is then calculated as follows:

4 $${\mathrm{SE}}\left( {\hat r_g} \right) = \sqrt {\frac{{B - 1}}{B}\mathop {\sum }\limits_{k = 1}^B \left( {\hat r_g - \hat r_g^{\left( k \right)}} \right)^2}$$

### Heterogeneity in SNP effects on complex traits

To test the difference in the SNP effects estimated in male or female datasets we applied a heterogeneity test. If $\hat \beta _m$ and $\hat \beta _f$ are the male and female per-allele effect estimates, and ${\mathrm{SE}}(\hat \beta _m)$ and ${\mathrm{SE}}(\hat \beta _f)$ are their corresponding standard errors, then we used the test statistic,

5 $$T_d = \frac{{\left( {\frac{1}{2}\hat \beta _m - \hat \beta _f} \right)^2}}{{\frac{1}{4}{\mathrm{SE}^2}\left( {\hat \beta _m} \right) + \mathrm{SE}^2}\left( {\hat \beta _f} \right)}$$

which follows a *χ*^2^-distribution with one degree of freedom under the null hypothesis of no difference in estimates under FDC assumption. We set a *P*-value threshold of *P*_Het_ < 5.0 × 10^–8^ to identify the markers with significant difference in estimated effects and further apply LD-clumping (*R*^2^ threshold of 0.05) to identify regions of heterogeneity. The coordinates of protein coding genes in these regions were extracted with BioMart tool (see URLs), using the genome assembly GRCh37.p13 from Genome Reference Consortium.

### Estimation of the SNP-heritability

We estimated the proportion of variance explained by X-chromosome SNPs in males and females separately using GREML and a genome partitioning approach as in ref. ^26^, which is implemented in the GCTA software package^18^. Here, we model the trait as,

6 $${\mathbf{y}} = {\mathbf{g}}_{\mathbf{G}} + {\mathbf{g}}_{\mathbf{X}} + {\mathbf{\varepsilon }}$$

where, y is a N × 1 vector of phenotype for each trait, with sample size *N*; **g**_**G**_ is an *N* × 1 vector of the total genetic effects from the autosome with ${\mathbf{g}}_{\mathbf{G}}\sim N(0,{\mathbf{A}}_{\mathbf{G}}\sigma _G^2)$ where **A**_**G**_ is the GRM between individuals estimated from 548,860 autosomal HapMap3 SNPs; **g**_**X**_ is an *N* × 1 vector of X-linked genetic effects with ${\mathbf{g}}_{\mathbf{X}}\sim N(0,{\mathbf{A}}_{\mathbf{X}}\sigma _X^2)$, where **A**_**X**_ is a GRM calculated from 253,842 X-chromosome SNPs; and ${\mathbf{\varepsilon }}\sim N(0,{\mathbf{I}}\sigma _e^2)$, is the residual. Partitioning in this way will allow for an estimation of the parameter *σ*^2^_*X*_ conditional on the autosomal GRM. Thus, we can estimate the proportion of phenotypic variance that is due to the X chromosome while controlling for sample structure captured by genetic variants on the autosome^26^. We applied this model to the 20 complex traits, limiting our analysis to a maximum of 100,000 unrelated males or females due to computational restrictions.

The standard errors of the M/F ratio of the estimated SNP-heritabilities on the X chromosome was estimated as,

7 $${\mathrm{SE}}^2 = \left( {\frac{{\hat h_m^2}}{{\hat h_f^2}}} \right)^2\left( {\frac{{\mathrm{SE}}^2\left( {\hat h_m^2} \right)}{{\left( {\hat h_m^2} \right)^2}} + \frac{{\mathrm{SE}}^2\left( {\hat h_f^2} \right)}{{\left( {\hat h_f^2} \right)^2}}} \right)$$

where $\hat h_m^2$ and $\hat h_f^2$ are the GREML-estimates of SNP-heritability in males and females, respectively, and ${\mathrm{SE}}(\hat h_m^2)$ and ${\mathrm{SE}}(\hat h_f^2)$ are the corresponding standard errors.

### Sex-stratified X-chromosome and autosomal *cis*-eQTL analysis

Gene expression levels were modelled as a linear function of the number of reference alleles for SNPs on the same chromosome in males and females, separately. We used GCTA^18^ and PLINK^35^ to analyse the CAGE and GTEx datasets, respectively. Sample structure was accounted for by adjusting for genotyping principal components and PEER factor in the GTEx analysis, and a random polygenic effect captured by an autosomal genetic relationship matrix in the CAGE analysis. For each gene expression probe/transcript, we identified the top associated SNP that satisfied a Bonferroni corrected significance threshold in the discovery sex (i.e. eQTL), and extracted the same eQTL in the other sex to compare the per-allele eQTL effect estimates between the sexes (see Estimation of dosage compensation coefficient (DCC), below).

### Sex differences in gene expression

Sex differences in X-linked gene expression in the CAGE dataset was examined with a mixed linear regression model implemented in GCTA^18^. We fit sex as a fixed effects covariate, and sample structure was accounted for with random polygenic effects captured by both an autosomal and X-linked genetic relationship matrix. A Wald statistic was used to assess significance, and a *P*-value (*P*_sex_diff_) was calculated by comparing the test statistic to a *χ*^2^-distribution with one degree of freedom.

### Summary data-based Mendelian randomisation (SMR)

The SMR and HEterogeneity In Dependent Instrument (HEIDI) tests^24^ are implemented in the SMR software package (see URLs). We applied the SMR method to summary-level GWAS data and the sex-stratified X-chromosome eQTL data generated in our analyses (UKB and CAGE, respectively) to test for pleiotropic associations between 1639 X-linked gene expression probes and 20 complex trait phenotypes. A total of 113, 66 and 136 probes with at least one *cis*-eQTL at GWS threshold *P*_eQTL_ < 5.0 × 10^–8^ were retained in male-, female- and in a combined male–female *cis*-SMR analysis, respectively. SMR analysis in the *trans* regions was performed with combined male–female data, with 78 probes with *trans*-eQTLs (*P*_eQTL_ < 5.0 × 10^–8^) included. A random sample of 100,000 unrelated UKB females of European ancestry was used as a reference for LD estimation. Trait-gene SMR associations were identified using a significance level of *P*_SMR_ < 3.0 × 10^−5^ (i.e. 0.05/1639). These associations were then tested for evidence of linkage, rather than pleiotropy/causality, using the HEIDI test, which tests for heterogeneity in the effect estimates of the exposure on the outcome at SNPs in LD with the top associated eSNP under the null hypothesis of no heterogeneity. Gene-trait associations with *P*_HEIDI_ > 0.05 were selected.

### Estimation of the effect size ratio

We refer to the effect size ratio as the ratio of M/F per-allele effect estimates for a single trait-SNP association. The corresponding standard errors are estimated as,

8 $${\mathrm{SE}}^2 = \left( {\frac{{\hat \beta _m}}{{\hat \beta _f}}} \right)^2\left( {\frac{{\mathrm{SE}^2}\left( {\hat \beta _m} \right)}{{\hat \beta _m^2}} + \frac{{\mathrm{SE}^2}\left( {\hat \beta _f} \right)}{{\hat \beta _f^2}}} \right)$$

As before, $\hat \beta _m$ and $\hat \beta _f$ are the M/F per-allele effect estimates, and ${\mathrm{SE}}(\hat \beta _m)$ and ${\mathrm{SE}}(\hat \beta _f)$ are the corresponding standard errors, respectively.

### Estimation of dosage compensation coefficient (DCC)

To compare the per-allele effect estimates across all conditionally independent trait-associated SNPs (complex trait analysis) and top eQTLs (gene expression analysis) identified in the discovery datasets, we calculated a DCC by regressing the per-allele effect estimates in males onto females weighted by inverse of the variance of male-specific estimates, and extracting the slope estimate and corresponding standard error. The estimates from sex-stratified XWAS, rather than joint effect estimates from the GCTA-COJO^21^ analysis were used for estimating DCC in the UKB traits. DCC is expected to take on values between 1 and 2, where DCC of 1 indicates that, on average, the effect sizes in males and females are equal (i.e. no DC or escape from XCI), and DCC of two indicates that, on average, the effect sizes in males are twice that of females (i.e. FDC).

### X-chromosome gene inactivation status

To determine X-chromosome inactivation status, we downloaded annotation from the “Reported XCI status” column in Supplementary Table 13 of^10^ and mapped gene expression probes to XCI status using the gene name. A total of 683 X-linked transcripts were available, where transcripts were classified as either Escape (82 transcripts), Variable (89 transcripts), Inactive (392 transcripts) or Unknown (120 transcripts). For each SNP in UKB dataset we determine if it is physically located within a gene to infer the presumable gene and its inactivation status for independent GWS SNPs. The CAGE and GTEx datasets were matched on the “Gene.name” and “Gene.ID” columns, respectively.

### Ethics

The research was carried out under the University of Queensland Institutional Human Research Ethics (UQ-HREC) Approval Number 2011001173 (UK biobank and GTEx human data) and UQ-HREC 2013000682 (CAGE human data). Each of the participating cohorts holds an individual ethics approval. For further details see: the UK Biobank (https://www.ukbiobank.ac.uk/the-ethics-and-governance-council/); the CAGE dataset;^23^ and the GTEx dataset^42^.

### URLs

For GTEx, see https://www.gtexportal.org/home/. For GCTA, see http://cnsgenomics.com/software/gcta/. For SMR, see http://cnsgenomics.com/software/smr/. For PLINK, see https://www.cog-genomics.org/plink2/. For BOLT-LMM, see https://data.broadinstitute.org/alkesgroup/BOLT-LMM/. For UK Biobank, see http://www.ukbiobank.ac.uk/. For BioMart, see http://grch37.ensembl.org/biomart/martview/.

### Reporting summary

Further information on research design is available in the Nature Research Reporting Summary linked to this article.

## Supplementary information

Supplementary information

Description of Additional Supplementary Files

Supplementary Data 1

Supplementary Data 2

Supplementary Data 3

Supplementary Data 4

Supplementary Data 5

Supplementary Data 6

Supplementary Data 7

Supplementary Data 8

Supplementary Data 9

Supplementary Data 10

Supplementary Data 11

Supplementary Data 12

Supplementary Data 13

Reporting Summary

## Data Availability

UK Biobank: The individual-level UK Biobank data is available upon application to the UK Biobank (http://www.ukbiobank.ac.uk/, accessed under project number 12514). Consortium for the Architecture of Gene Expression (CAGE): As per the ethics agreement of the CAGE consortium, all raw and normalized genotype and expression data are available to consortium members. Consortium membership is open, but requires approval from the steering committee. GTEx: The fully-processed, normalised and filtered RNA-seq GTEx v6p data were downloaded from the GTEx Portal (https://www.gtexportal.org/home/datasets) along with corresponding covariate files. X-chromosome imputed SNP data was obtained from dbGap (Accession phs000424.v6.p1). Data generated in this study, including full summary statistics for XWAS on 20 complex traits from the UKB, and eQTL results from CAGE and GTEx, have been deposited at: http://cnsgenomics.com/data.html
