# Supplementary material for: The effect of X-linked dosage compensation on complex trait variation
Source: Nat Commun. 2019 Jul 8;10:3009. doi: 10.1038/s41467-019-10598-y (PMC6614401; doi:10.1038/s41467-019-10598-y)
Supplement: Supplementary file 1 — Supplementary information [file 41467_2019_10598_MOESM1_ESM.pdf]

Supplementary Information

The effect of X-linked dosage compensation on complex trait variation

Julia Sidorenko[1*], Irfahan Kassam[1*], Kathryn E. Kemper[1], Jian Zeng[1], Luke R. Lloyd-Jones[1], Grant W. Montgomery[1], Greg Gibson[2], Andres Metspalu[3], Tonu Esko[3], Jian Yang[1,4], Allan F. McRae[1†], Peter M. Visscher[1,4†]

[1]Institute for Molecular Bioscience, The University of Queensland, Brisbane, Australia

[2]School of Biology and Centre for Integrative Genomics, Georgia Institute of Technology, Atlanta, United States of America

[3]Estonian Genome Centre, Institute of Genomics, University of Tartu, Tartu, Estonia

[4]Queensland Brain Institute, The University of Queensland, Brisbane, Australia

[*]These authors contributed equally to this work

[†]These authors jointly supervised this work

Correspondence: peter.visscher@uq.edu.au, j.sidorenko@imb.uq.edu.au

Supplementary Note 1

Comparison of the X chromosome to the autosomes

For complex traits such as educational attainment (EA), height or BMI, the proportion of genetic variation due to the X chromosome is reported to be lower than expected for an autosome of similar length[1,2]. The genetic variance contributed by X-chromosome SNPs is proportional to $\sum var(x) * \beta^2$, where $var(x)$ is the variance of the SNP allele counts, x, and β is the per-allele effect size at trait loci. In this study we have established that the SNP-heritability (h^2_{SNP}) attributable to the X chromosome is on average 0.6% in males and 0.3% in females across 20 complex traits in the UK Biobank (Supplementary Table 2), indicating that dosage compensation mechanisms on the X chromosome contribute to the difference in the X-linked genetic variances between males and females. Therefore, the dosage compensation in females is one factor that may downwardly bias the overall X-linked heritability estimates across sexes. We chose height and BMI, two traits with significant X-linked heritability, to evaluate the relationship between the autosomes and the X chromosome in males. When compared to the autosomal per-chromosome estimates of h^2_{SNP}, the X-specific h^2_{SNP} estimates were close to the those of the chromosomes with length <100Mb (Supplementary Figure 1A). To further disentangle whether the lower than expected amount of X-linked variance is attributable to the haploidy in males or on average smaller per-allele effect of the associated loci, we evaluated the number of trait-associated loci. To achieve similar power of detecting trait-associated variants, we performed autosomal association analysis in the set of males down-sampled by a factor of two, and selected independently associated SNPs using GCTA-COJO [3] algorithm.

As expected in the case of similar effect size between autosomes and the X-chromosome, we did not detect fewer X-linked SNPs for height and BMI, but rather the number of identified loci was close to the largest autosomes (Supplementary Figure 1B). We observed no significant difference in means between the absolute values of the autosomal and the X-chromosomal effect sizes of the trait-associated loci for both traits (P=0.56 and P=0.58 for height and BMI, respectively) (Supplementary Figure 1C). Therefore, our results indicate that the smaller number of X-linked loci identifiable in a GWAS for a given sample size and the smaller X-linked h^2_{SNP}, are attributable to haploidy in males and random X-chromosome inactivation in females.

Supplementary Note 2

Evidence for hormonally influenced sex-specific genetic control in the four regions of heterogeneity on the X chromosome

We observed a highly significant trait association in males and lack of association in females in the heterogeneity region 1 (Xp22.31) for 5 traits: body fat percentage (Fat%), haemoglobin concentration (Hgb), haematocrit percentage (Hcrit), red blood cell count (RBC) and heel bone

mineral density (hBMD) (Figure 2). Notably, this region near the *FAM9A/FAM9B* genes, has been shown to be significantly associated with male-specific traits such as testosterone levels [4], male pattern baldness [5,6] and age at voice drop [6]. Moreover, the *FAM9A/FAM9B* genes have been shown to be expressed exclusively in testis in hybridization experiments [7]. Indeed, in the GTEx data (see URLs), we found that *FAM9A* is highly expressed in testis only, with lower levels of expression of *FAM9B* in both uterus and testis, supporting the male-specific architecture for this locus and suggesting the androgenic pathway. Androgens play essential erythropoiesis promoting- [8], fat-reducing- [9] and anti-osteoporotic- [10] roles. Thus, we presume that a pleiotropic effect of the heterogeneity region 1 on erythropoiesis associated traits (Hgb, Hcrit and RBC), Fat% and hBMD, may be mediated by androgen levels.

The *NROB1* gene in the heterogeneity region 2 (Xp21.2), which encodes the DAX1 protein, was a candidate gene for male-specific genetic control for height in this region (Figure 2). DAX1 is essential for regulation of hormone production and loss of DAX1 function leads to adrenal insufficiency and hypogonadotropic hypogonadism [11]. Moreover, Xp21.2 region in known as a dosage-sensitive sex reversal region, where its duplication or deletion is associated with male-female or female-male sex reversal [12–14].

The top signal in the heterogeneity region 4 was located in another well-known androgen-associated locus (Xq12) near the androgen receptor (*AR*) gene (Figure 2). The significant heterogeneity in this region between males and females for Fat% supports the male-specific fat-reducing effect of androgens. Notably, we observed the sex-specific heterogeneity in regions 1 and 4 for Fat% but not for BMI, suggesting that, although highly correlated, these traits differ in aetiology.

For Hgb and Hcrit, the main heterogeneity signal was identified in Xp11.21 (heterogeneity region 3) (Figure 2). This region is shown to be associated with blood zinc concentrations (near *KLF8*, *ZXDA* and *ZXDB* encoding Zn-finger proteins [15]) and male-pattern baldness [6]. Zinc has been shown to modulate serum testosterone levels in men [16] and is associated with haemoglobin concentrations in epidemiological studies [17]. However, we find that the 5' end of the region 3 is adjacent to the *ALAS2* gene, encoding a protein involved in heme synthesis and thus erythropoiesis (OMIM *301300). Mutations in this gene cause sideroblastic anaemia with X-linked recessive inheritance (OMIM #300751). Thus, the evidence for the androgen-dependent effect of this region remains inconclusive.

To further support the male-specific architecture for the heterogeneity regions, we sought to determine if genes in these regions may be androgen-responsive. None of these genes were shown to be androgen-regulated [18]. However, the *KLF4, KLF5*, *KLF10* and *KLF13* genes from the Krüppel-like factor (KLF) family are reported to be androgen-dependent [18]. Notably, *KLF8*, a gene located in the Xp11.21 region is shown to repress the *KLF4* transcription [19], where the *KLF4* and *AR* genes appear to be reciprocally upregulated [20]. Additionally, a burden of rare mutations in the *KLF1,* encoding a transcriptional activator of the *KLF8* [21], is also significantly associated with blood related traits, such as red blood cell distribution width and mean

corpuscular haemoglobin [22]. This suggests that the *KLF8* could be implicated in the male-specific architecture of the heterogeneity region 3 for Hgb and Hcrit.

Supplementary Note 3

***ITM2A* gene-trait association results support the *ITM2A* being the subject to inactivation**

Variants near *ITM2A* were shown to be associated with height in a previous study [23] and with height, BMR, Grip, WHR and FEV1 in the current XWAS analysis. In the combined male-female SMR [24] analysis we also observed evidence for pleiotropic association ($P_{SMR}<3.0x10^{-5}$) of the *ITM2A* expression (tagged by probe ILMN_2076600) with 6 traits: height, BMR, Grip, WHR, FEV1 and RBC (genetic instrument SNP rs10126553, Supplementary data 12). However, only associations corresponding to RBC pass the test for heterogeneity ($P_{HEIDI}>0.05$), which aims to distinguish pleiotropy/causality from linkage. For the remaining traits, P_{HEIDI} varied from $6.5x10^{-3}$ for WHR to $8.0x10^{-16}$ for height, indicating heterogeneity in gene expression effect on the trait estimated at different eSNPs that are in LD with the top-associated eSNP. That is, we cannot reject the null hypothesis that the gene-trait association is due to a single genetic variant. SMR analysis in *trans* regions on the X chromosome identified additional associations between the expression of the *ITM2A* gene and height and BMR, which was mediated by a *trans*-eQTL located 2.2Mb upstream the *ITM2A* (SNP rs112933714, Supplementary data 13). The mean male-female effect size ratio for the genetic instrument, SNP rs10126553 ($P_{eQTL,combined}=1.5x10^{-76}$), across these 7 traits (not filtered on P_{HEIDI} value) was 1.83 (SD=0.25) (Supplementary data 12), and 2.30 (SD=0.65) for the *trans* acting variant rs112933714 across two traits with significant *trans*-eQTLs (Supplementary data 13), in agreement with the reported Inactive status of the *ITM2A* gene.

Supplementary Figures

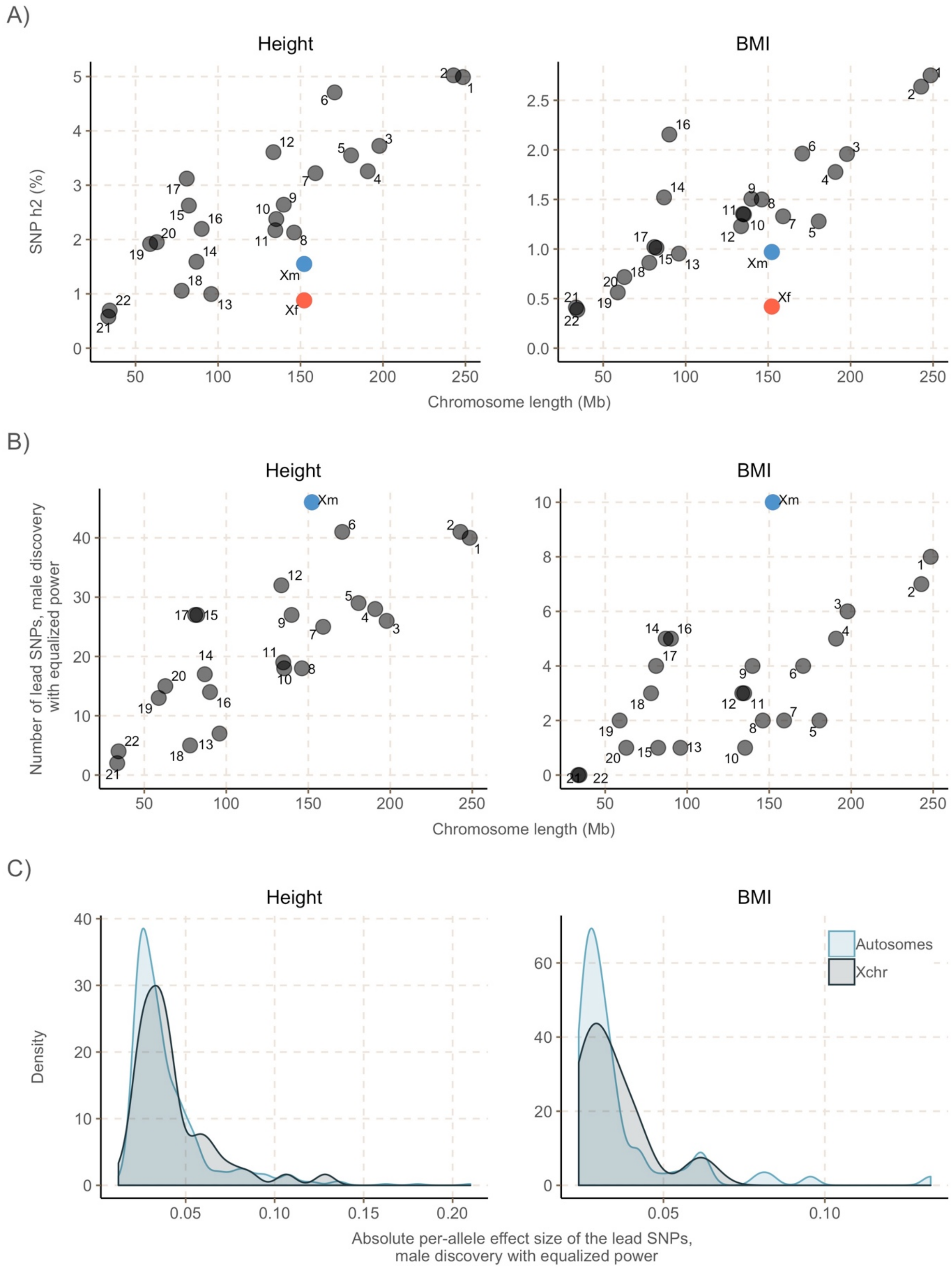

Supplementary Figure 1. Comparison of the X chromosome to the autosomes. (A) The per-chromosome SNP-heritability and (B) the number of lead SNPs (male discovery, minor allele frequency >1%) for height and body mass index (BMI) are compared to the physical length of the chromosome. We used GCTA-GREML [25] to estimate the per-chromosome SNP-

heritability in a sample of 50,000 unrelated males in the autosomal analysis and 100,000 unrelated males and females in the X-chromosome analysis. The lead SNPs were identified using GCTA-COJO [3], using the summary statistics from the autosomal association analyses conducted in a sample of ~104,000 males and the X-chromosomal analysis in ~208,000 males. The distribution of the per-allele effect estimates of these autosomal and X-chromosomal lead SNPs are compared in panel (C).

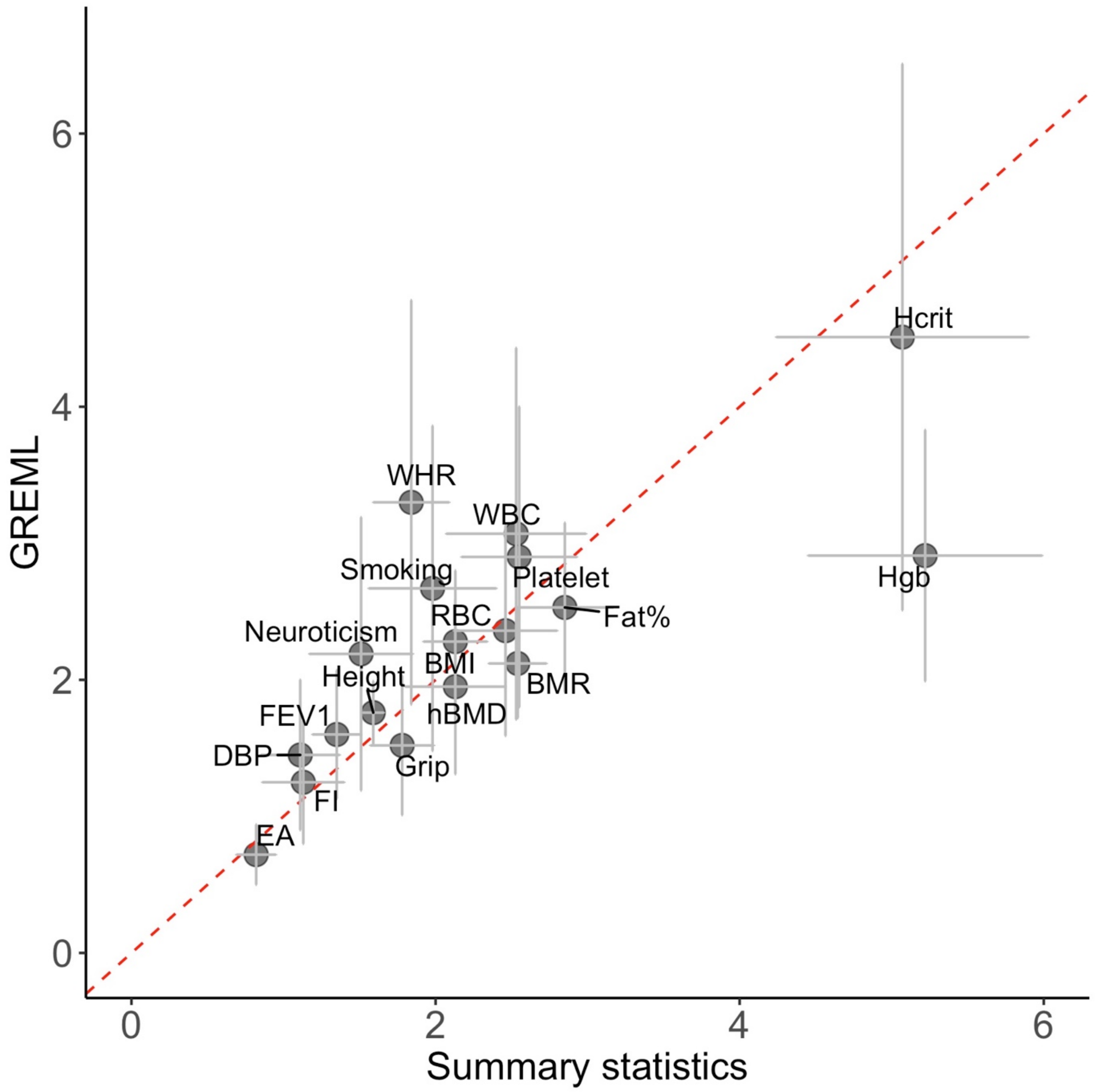

Supplementary Figure 2. Dosage compensation (DC) ratio estimates from summary statistics and GCTA-GREML. The male-female SNP-heritability ratios were compared for 18 traits with significant X-linked SNP-heritability estimates in both sexes (from GREML-analysis). The red dashed line indicates the expected correlation of 1. The bars represent the standard error. **Traits:** standing height (Height), forced expiratory volume in 1-second (FEV1), smoking status (Smoking), hand grip strength, right (Grip), body mass index (BMI), body fat percentage (Fat%), basal metabolic rate (BMR), waist to hip ratio (WHR), diastolic blood pressure (DBP), heel bone mineral density T-score (hBMD), fluid intelligence score (FI), neuroticism score (Neuroticism), educational attainment (EA), white blood cell (leukocyte) count (WBC), platelet count (Platelet), red blood cell (erythrocyte) count (RBC), haemoglobin concentration (Hgb), Haematocrit percentage (Hcrit).

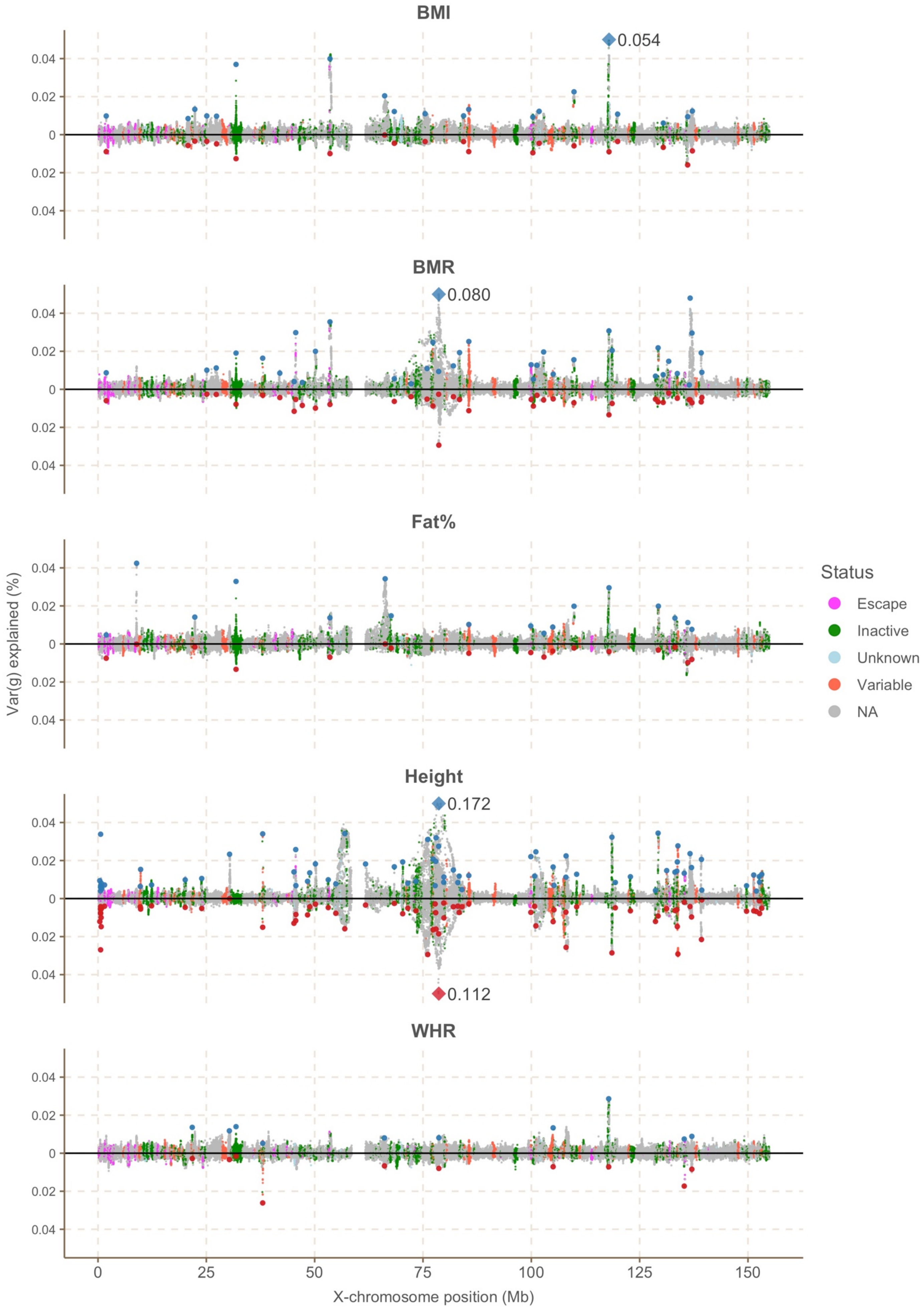
BMI
0.054
BMR
0.080
Fat%
Height
0.172
0.112
WHR
Var(g) explained (%)
X-chromosome position (Mb)
0.04
0.02
0
0
25
50
75
100
125
150
Status
Escape
Inactive
Unknown
Variable
NA

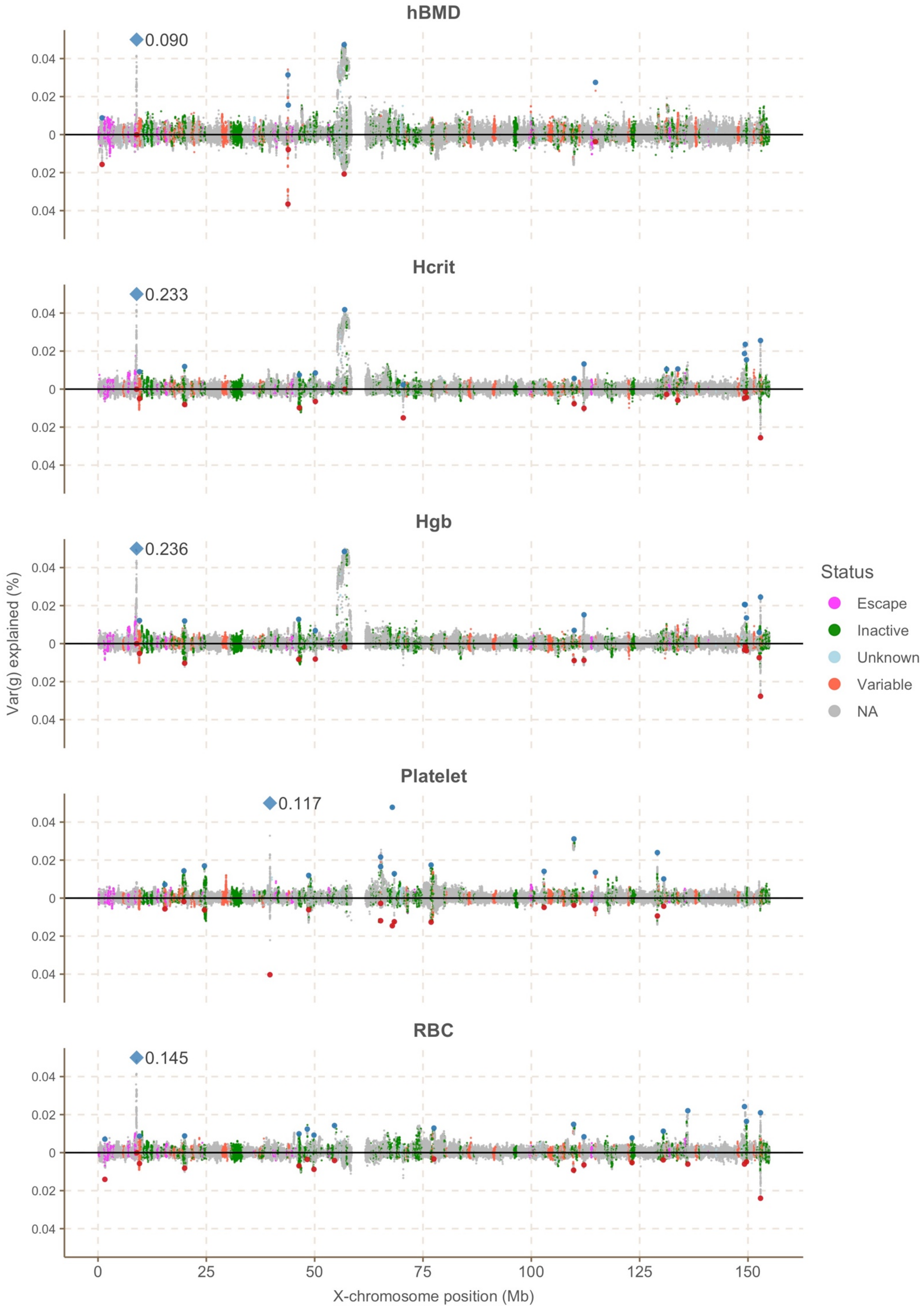

hBMD
0.090
Hcrit
0.233
Hgb
0.236
Platelet
0.117
RBC
0.145
Var(g) explained (%)
X-chromosome position (Mb)
0.04
0.02
0
0.02
0.04
0
25
50
75
100
125
150
Status
Escape
Inactive
Unknown
Variable
NA

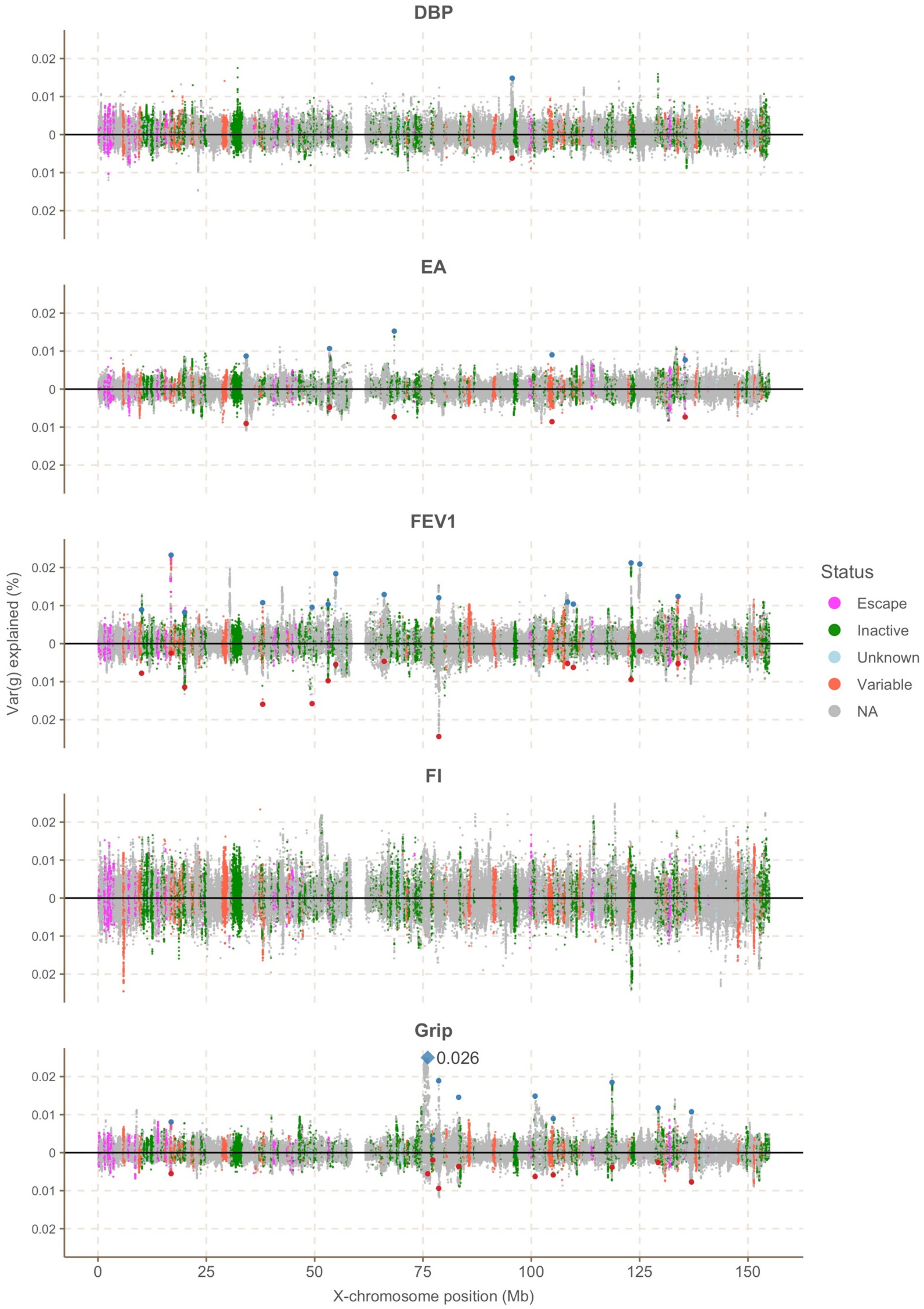
DBP
EA
FEV1
FI
Grip
0.026
Var(g) explained (%)
0.02
0.01
0
X-chromosome position (Mb)
0
25
50
75
100
125
150
Status
Escape
Inactive
Unknown
Variable
NA

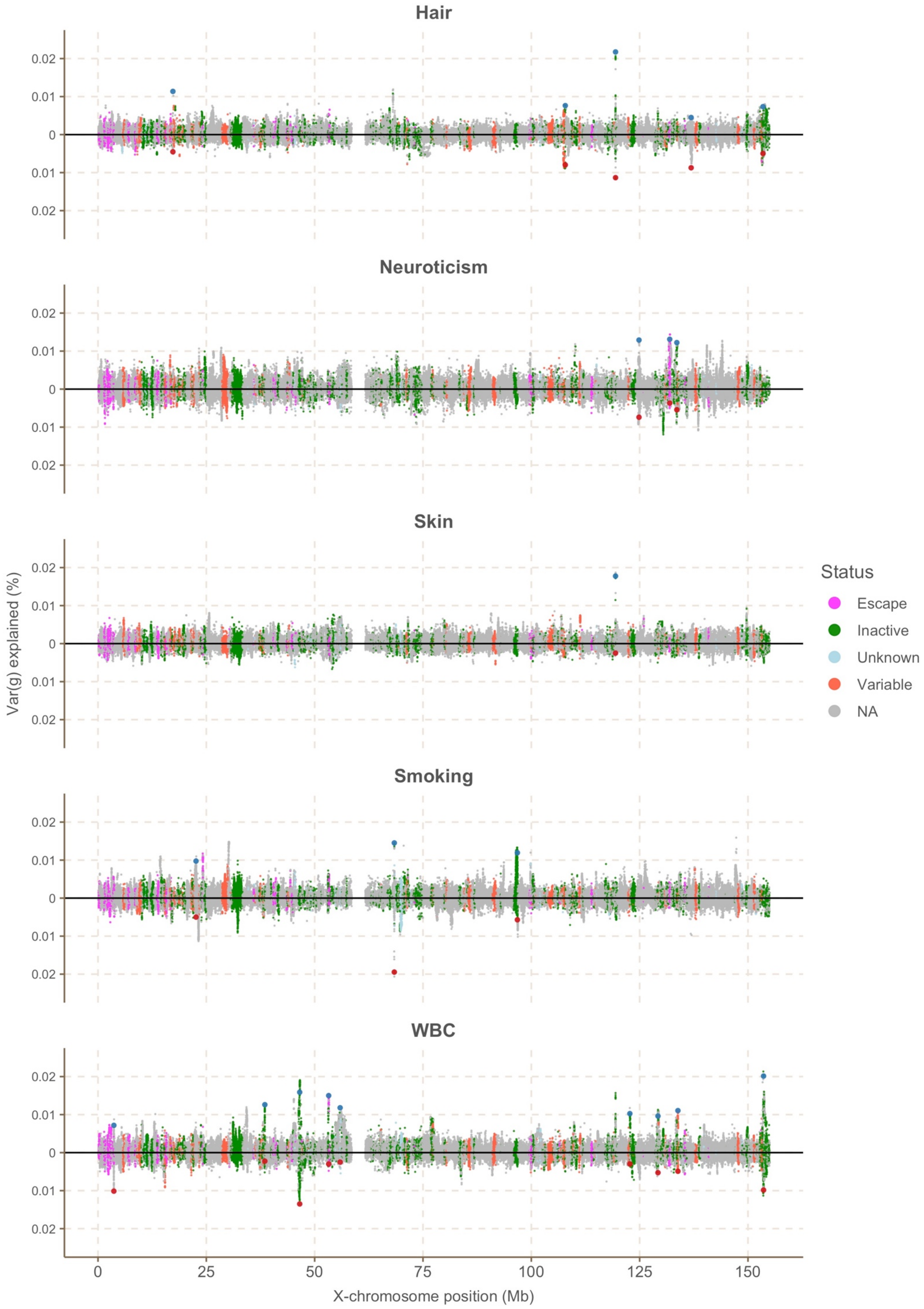

Hair
Neuroticism
Skin
Smoking
WBC
0.02
0.01
0
0.01
0.02
Var(g) explained (%)
0
25
50
75
100
125
150
X-chromosome position (Mb)
Status
Escape
Inactive
Unknown
Variable
NA

Supplementary Figure 3. Sex-specific variance explained on the X chromosome. The male-specific estimates are presented on the top of each plot and female-specific estimates are on the bottom, with the variance of the lead SNPs (selected in the combined COJO-GCTA analysis) highlighted by larger blue circles (males) and red circles (females). Genetic variance contributed by the SNP in each sex was calculated as $var_m = p(1-p)\beta_m^2$ and $var_f = 2p(1-p)\beta_f^2$ in the non-PAR region for males and females, respectively, and $2p(1-p)\beta^2$ in the PAR region for either sex. β_m, β_f and β are the male-, female-, and either male- or female-specific per-allele effect estimates from our sex-stratified XWAS analysis; p is the minor allele frequency. For better scalability and to ease visualisation, the scale on the y-axis is truncated either at the value of 0.05 or 0.025. The diamonds represent the truncated loci and the maximum estimates for those loci are presented. The base pair positions are coloured according to the reported inactivation status [26]. **Traits:** standing height (Height), forced expiratory volume in 1-second (FEV1), smoking status (Smoking), hand grip strength, right (Grip), body mass index (BMI), body fat percentage (Fat%), basal metabolic rate (BMR), waist to hip ratio (WHR), diastolic blood pressure (DBP), heel bone mineral density T-score (hBMD), fluid intelligence score (FI), neuroticism score (Neuroticism), educational attainment (EA), skin colour (Skin), hair colour (Hair), white blood cell (leukocyte) count (WBC), platelet count (Platelet), red blood cell (erythrocyte) count (RBC), haemoglobin concentration (Hgb), Haematocrit percentage (Hcrit).

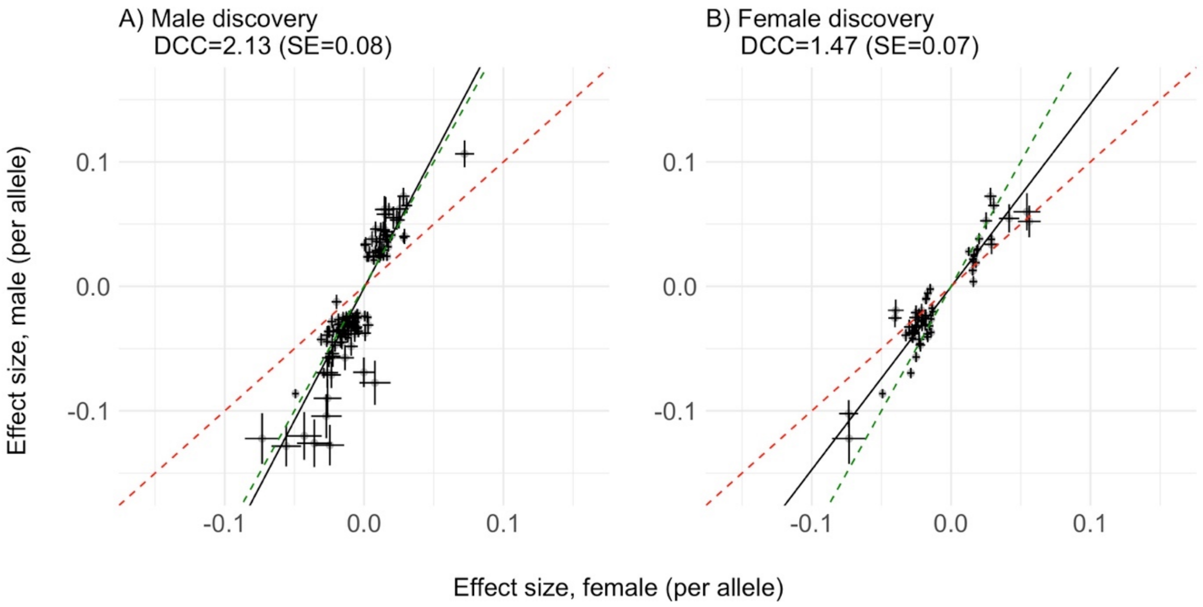

Supplementary Figure 4. Dosage compensation coeffieicent (DCC) in complex traits analysis, non-pseudoautosomal region (non-PAR). Comparison of the male- and female-specific per-allele effect estimates (+/- SE) for the lead SNPs identified in the A) male discovery set (*M*=143 SNPs) or B) female discovery set (*M*=61 SNPs). The SNPs located in the regions of heterogeneity are excluded. The green and red dashed lines indicate the expectations under full DC and escape from X-inactivation, respectively. The black line represents DCC. SE, standard error.

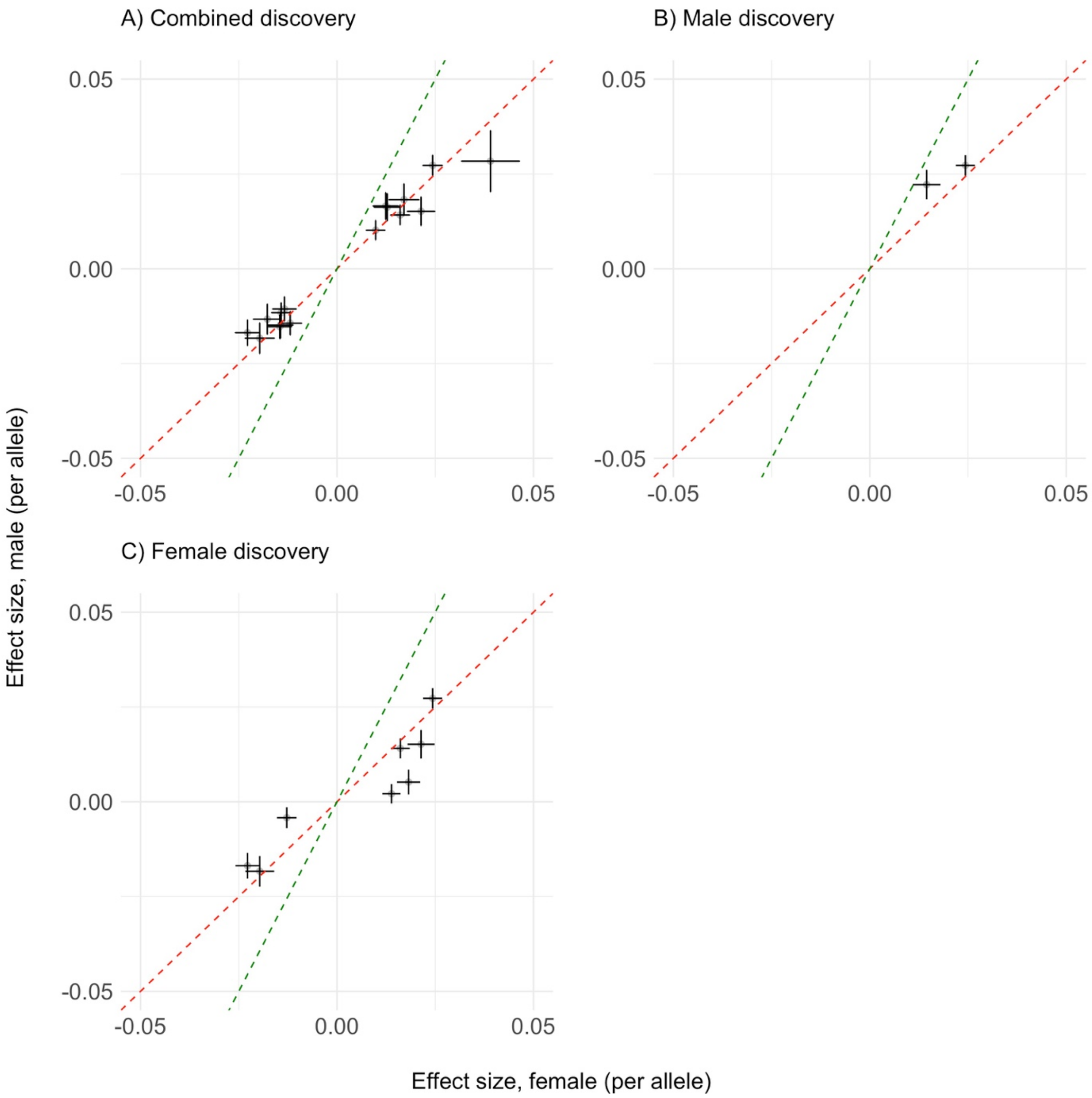

Supplementary Figure 5. Dosage compensation coeffieicent (DCC) in complex traits analysis, pseudoautosomal region (PAR). Comparison of per-allele effects from sex-specific analyses (+/- SE) of lead SNPs in as identified in a A) combined discovery set (*M*=16 SNPs), B) male discovery set (*M*=2 SNPs) or C) female discovery set (*M*=8 SNPs). The green and red dashed lines indicate the expectations under full DC and escape from X-inactivation, respectively. DCC was not estimated due to low number of lead SNPs in PAR.

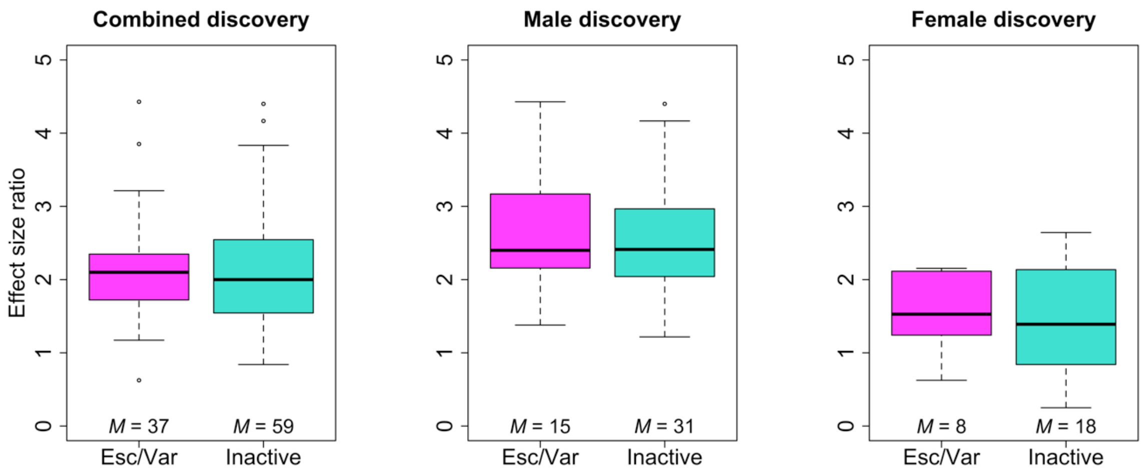

Supplementary Figure 6. Effects size ratios in complex traits analysis, non-pseudoautosomal region (non-PAR). Effects size ratios for the lead SNPs across the analysed complex traits are compared between Escape/Variable and Inactive groups, which include SNPs physically located within a gene region with previously reported XCI status[26]. We exclude variants in the regions of heterogeneity as well as 2 variants with the absolute ratio values > 10 (male discovery sample). *M*, number of SNPs. Center line represents median; box limits represent upper and lower quartiles; whiskers represent 1.5x interquartile range; individual points are outliers.

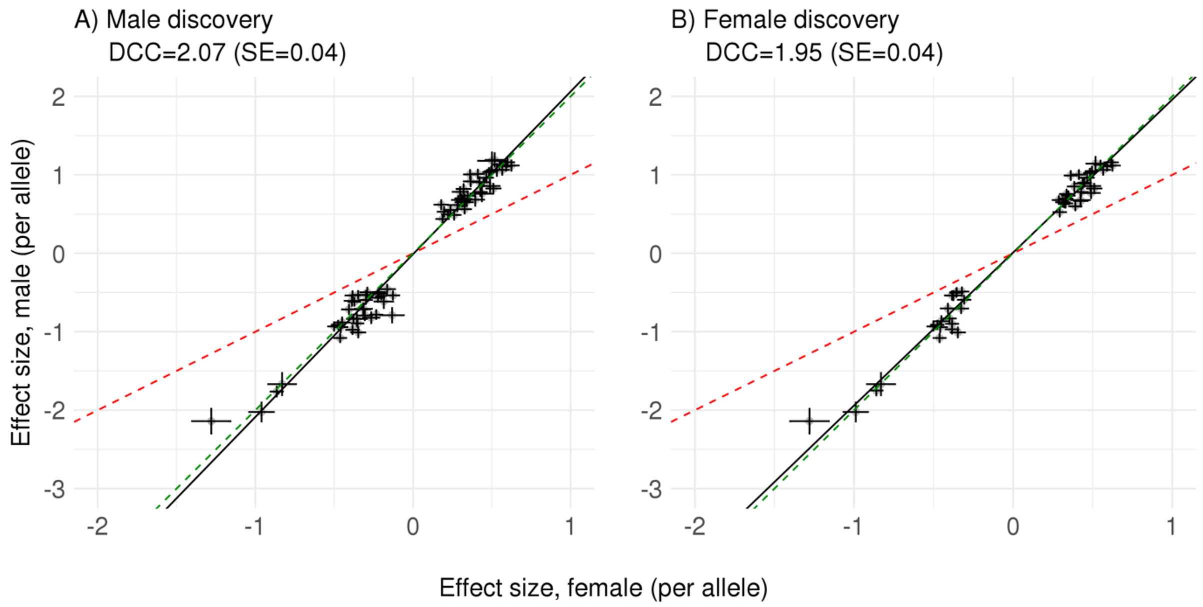

Supplementary Figure 7. Dosage compensation coeffieicent (DCC) in CAGE whole blood, non-pseudoautosomal region (non-PAR). Comparison of per-allele effects from sex-specific analyses (+/- SE) for X-chromosome *cis*-eQTLs in CAGE whole blood. DCC of 1.95 (SE=0.04) is observed for 51 eQTLs (P_{eQTL}<1.6x10^{-10}) in the female discovery analysis, and DCC of 2.07 (SE=0.04) for 74 eQTLs (P_{eQTL}<1.6x10^{-10}) in the male discovery analysis. The green and red dashed lines indicate the expectations under full DC and escape from X-inactivation, respectively. The black line represents DCC. SE, standard error

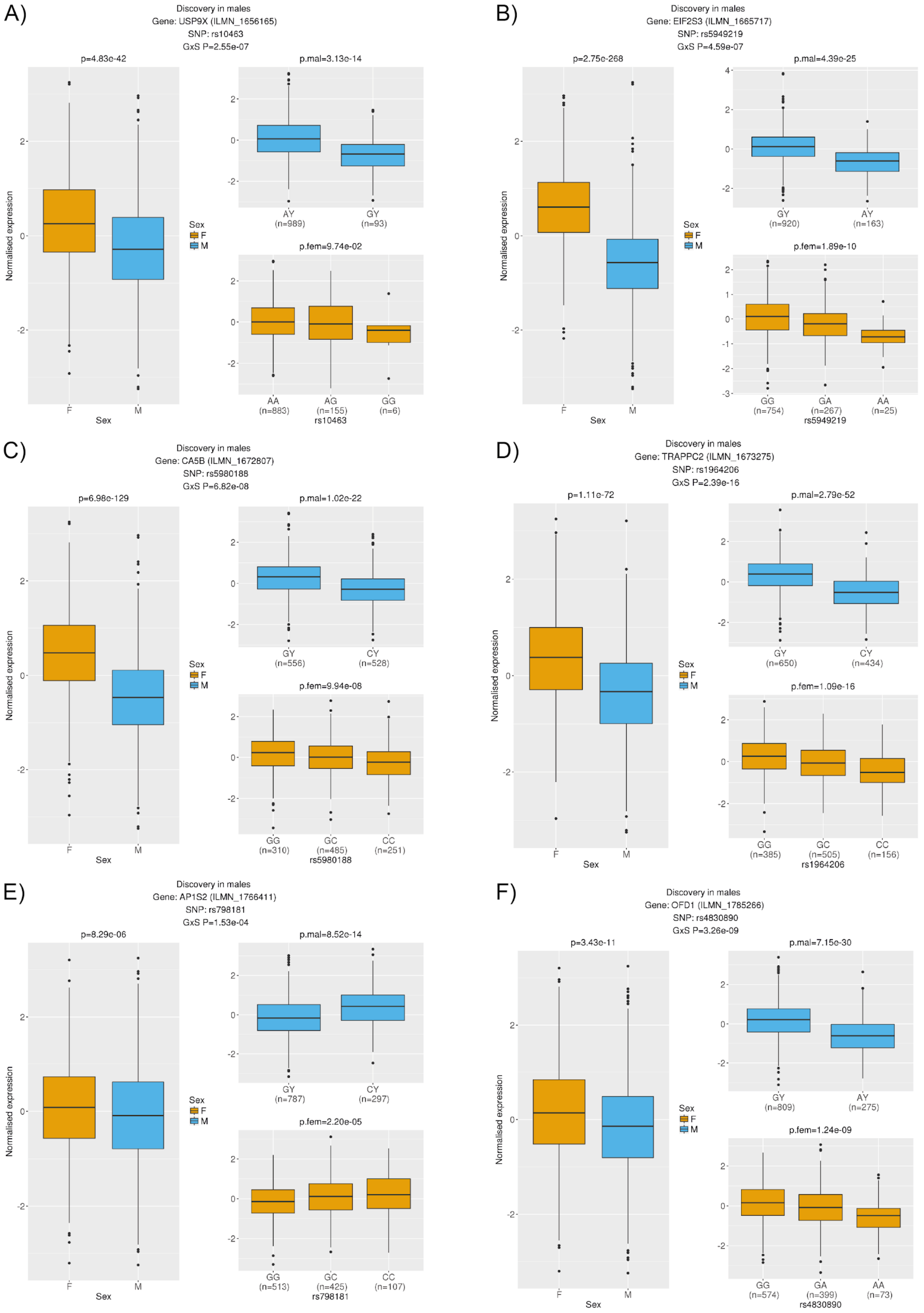

Supplementary Figure 8. Sex differences in X-chromosome gene expression may not be due to escape from XCI. A total of 6 eQTLs identified in the male discovery *cis*-eQTL analysis in CAGE whole blood are annotated to escape XCI. These genes show higher

expression in females compared to males (P_{sex_diff}<3.1x10^{-3}, i.e. 0.05/16), as expected for genes that escape from XCI, but also significant differences between the effect estimate of the top associated SNP on gene expression after correction for mean differences in expression between the sexes (genotype-by-sex interaction P_{GxS}<3.1x10^{-3}), which is consistent with FDC. This suggests that sex differences in the expression of these genes may not be due to escape from XCI. Orange corresponds to females. Blue corresponds to males. Center line represents median; box limits represent upper and lower quartiles; whiskers represent 1.5x interquartile range; individual points are outliers.

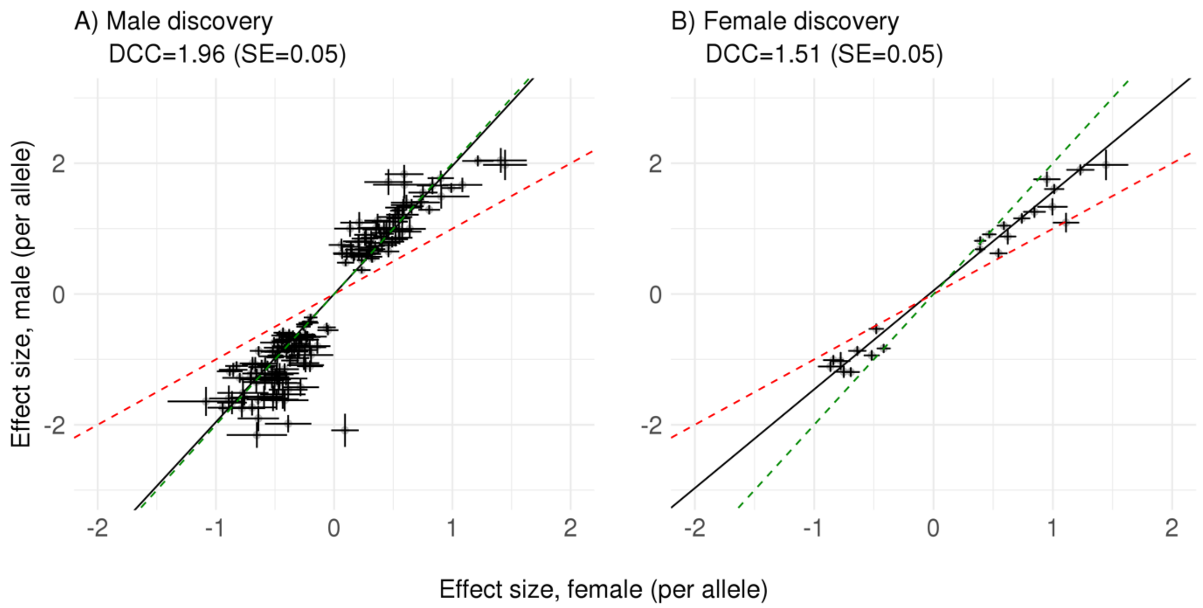

Supplementary Figure 9. Dosage compensation coeffieicent (DCC) across 22 tissues in GTEx, non-pseudoautosomal region (non-PAR). The per-allele effect estimates of top eQTLs across all 22 tissues in GTEx in the discovery sex is compared to the corresponding eQTL in the other sex from the matching tissue. DCC of 1.96 (SE=0.05) is observed for 175 eQTLs in the male discovery analysis, and 1.51 (SE=0.05) for 23 eQTLs in the female discovery analysis. The green and red dashed lines indicate the expectations under full DC and escape from X-inactivation, respectively. The black line represents DCC. SE, standard error.

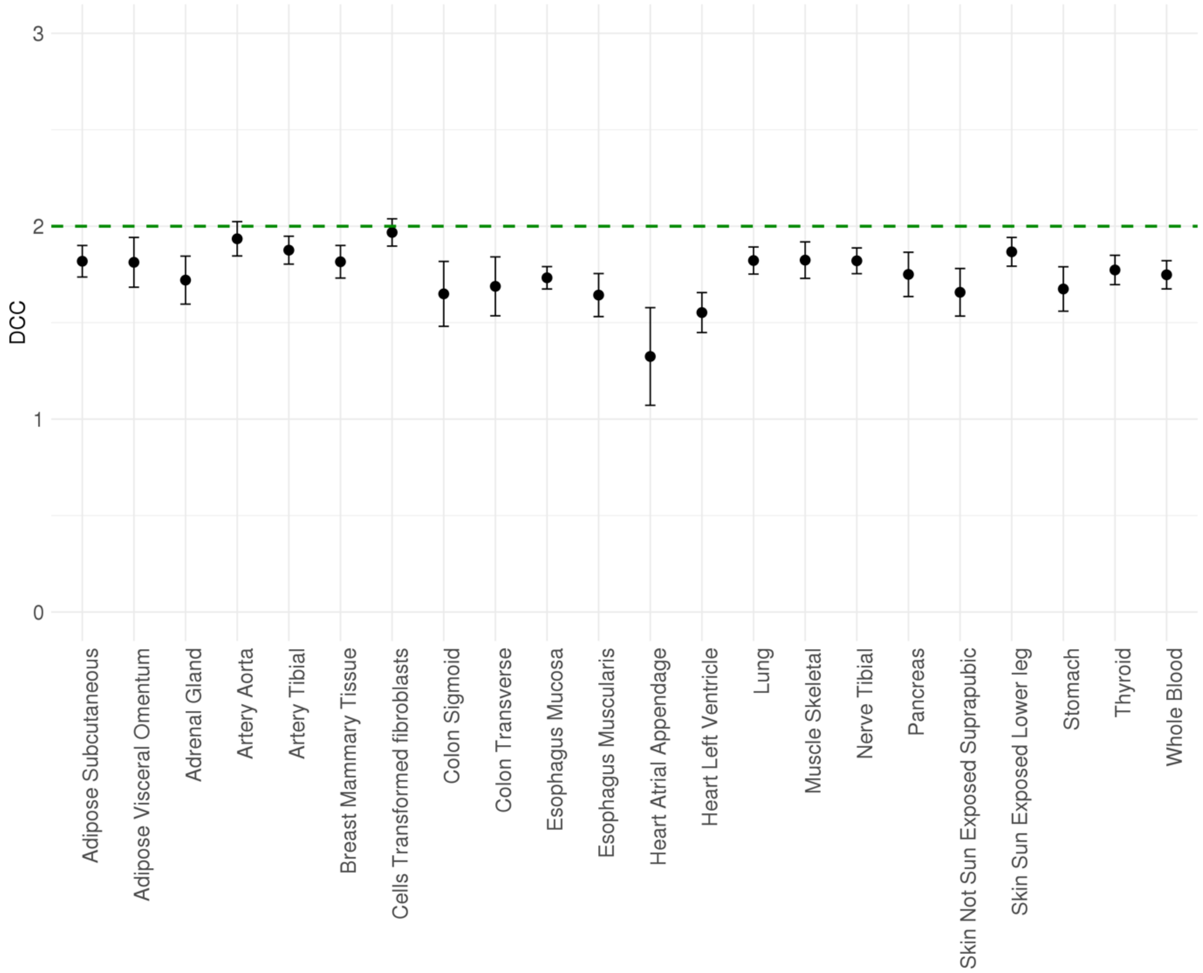

Supplementary Figure 10: Dosage compensation coeffieicent (DCC) for eQTLs from an interaction eQTL analysis across tissues. An eQTL interaction analysis of males and females combined identified a mean of 41 eQTLs (SD=20) that satisfied a within tissue Bonferroni significance threshold across the 22 tissues. This gave a mean DCC of 1.75 (SD=0.14) across the 22 tissue. The green dashed line indicates the expectation under full dosage compensation model. The bars represent the standard error. SD, standard deviation.

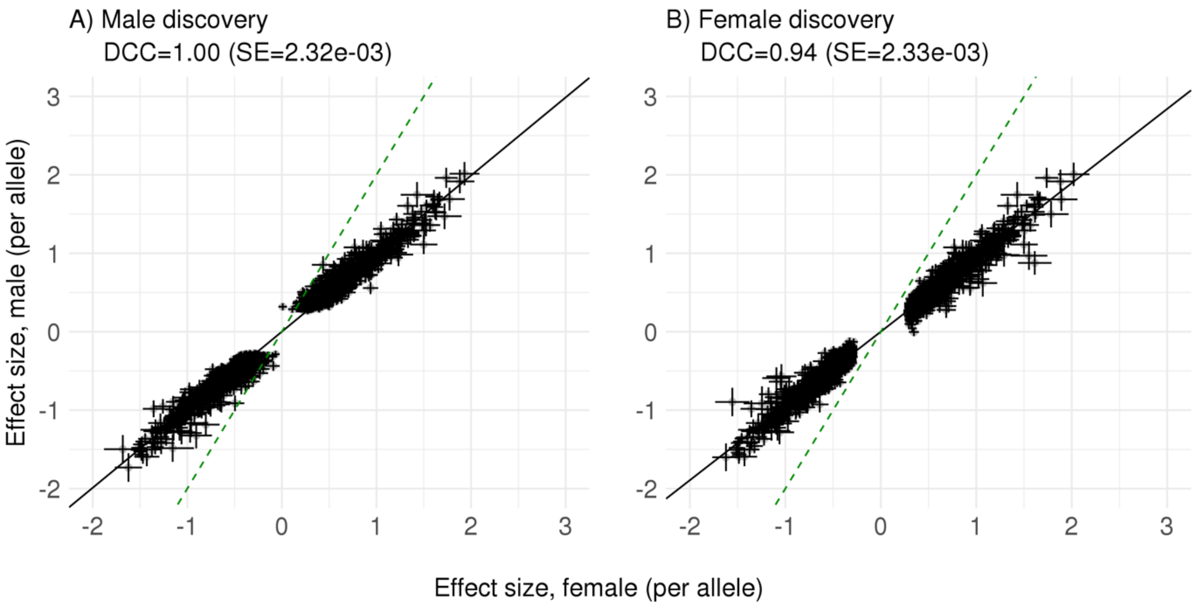

Supplementary Figure 11. Autosomal dosage compensation coefficient (DCC) in CAGE whole blood. Comparison of per-allele effects from sex-specific analyses (+/- SE) for autosomal *cis*-eQTLs identified in CAGE whole blood. DCC is expected to be equal in males and females. DCC of 1.00 (SE=$2.3x10^{-3}$) is observed for 3,116 eQTLs with $P_{eQTL}<10^{-10}$ in the male discovery analysis, and 0.94 (SE=$2.3x10^{-3}$) for 3,165 eQTLs with $P_{eQTL}<10^{-10}$ in the female discovery analysis. The green dashed line represents the $y=2x$ line. The black line represents DCC. SE, standard error

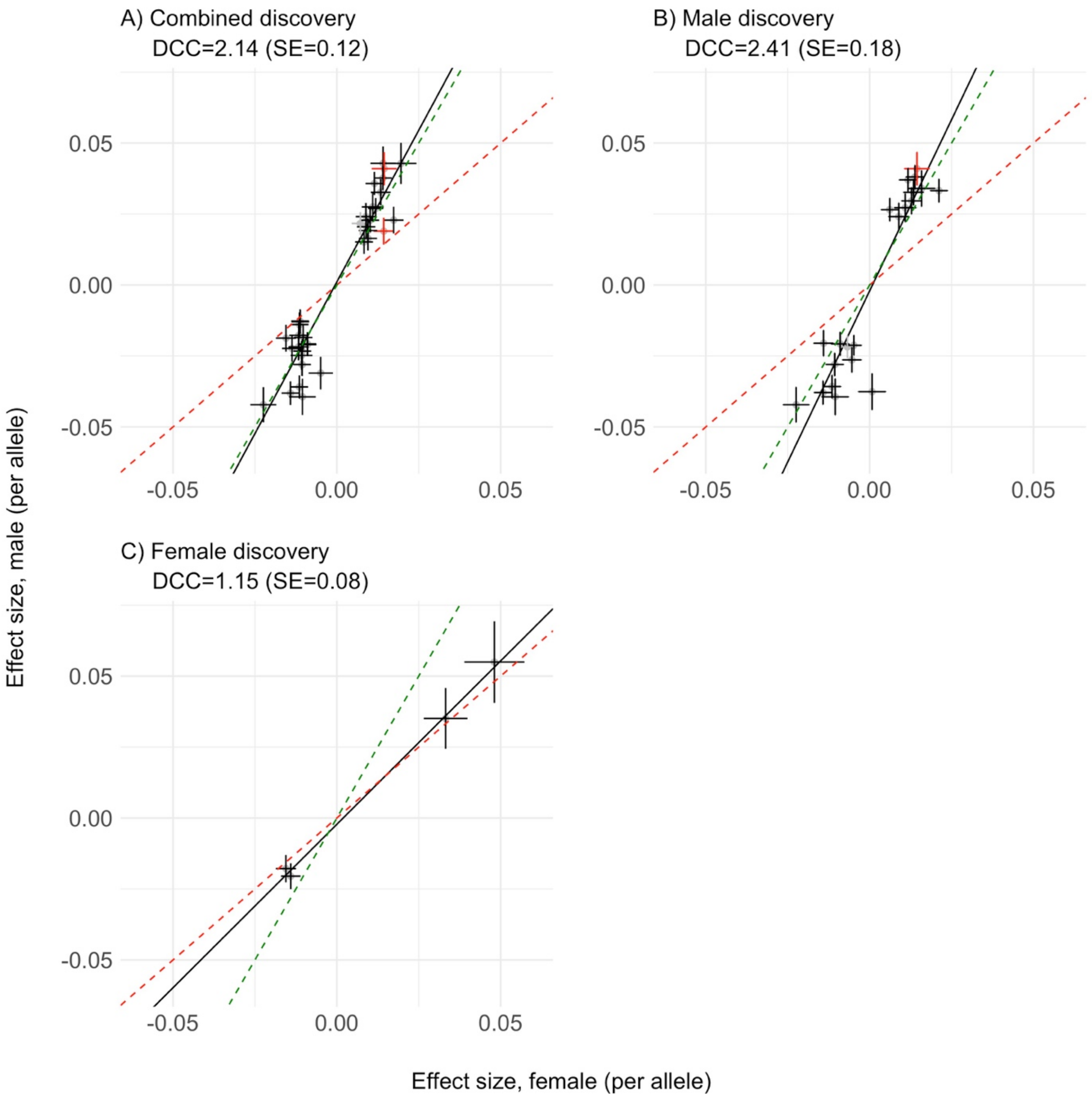

Supplementary Figure 12. Dosage compensation coeffieicent (DCC) for SMR hits, non-pseudoautosomal region (non-PAR). Comparison of per-allele effects from sex-specific analyses (+/- SE) of the SNPs associated with complex traits through gene expression, as identified in a A) combined male-female SMR analysis (*M*=36 SNPs), and sex-stratified SMR analyses (B, *M*=23 SNPs; C, *M*=4 SNPs). The SNPs are coloured according to the reported inactivation status of the genes that showed evidence of pleiotropic association with the phenotypic traits (SMR genes, red = Escape/Variable, black = Inactive, grey = Unknown). The SMR results are presented in the Supplementary data 9-11. The green and red dashed lines indicate the expectations under full DC and escape from X-inactivation, respectively. The black line represents DCC. SE, standard error.

Supplementary Tables 1-6

Supplementary Table 1. A) The UK Biobank trait information

				Male					Female					Total
**Trait**	**Abbreviation**	**UKB identifier**	**Covariates***	***N***	**Min.**	**Mean**	**Max.**	**SD[#]**	***N***	**Min.**	**Mean**	**Max.**	**SD[#]**	***N***
Standing height (cm)	Height	50-0.0-50-2.0	Age, age^2	207,920	139.00	175.84	209.00	6.64	246,694	126.00	162.63	199.00	6.10	454,614
Forced expiratory volume in 1-second, Best measure (litres)	FEV1	20150-0.0	Age, age^2	158,692	0.23	3.35	7.67	0.66	183,353	0.09	2.43	5.57	0.46	342,045
Smoking status	Smoking	20116-0.0	YOB (as factor)	207,536	0.00	0.52	1.00	0.49	246,155	0.00	0.41	1.00	0.49	453,691
Hand grip strength (right, kg)	Grip	47-0.0 - 47-2.0	Age, age^2	207,696	0.00	40.42	90.00	8.82	246,112	0.00	24.34	58.00	6.17	453,808
Body mass index ($kg\ m^{-2}$)	BMI	21001-0.0 - 21001-2.0	Age, age^2	207,649	12.81	27.84	53.28	4.21	246,387	12.12	27.01	57.85	5.10	454,036
Body fat percentage (%)	Fat%	23099-0.0 - 23099-1.0	Age, age^2	204,362	5.00	25.29	54.70	5.70	243,302	6.00	36.53	69.80	6.78	447,664
Basal metabolic rate (kJ)	BMR	23105-0.0 - 23105-1.0	Age, age^2	204,584	3883	7795.67	13975	1006	243,226	3531	5648.26	9644	651.96	447,810
Waist to hip ratio	WHR	NA^	Age, age^2	207,878	0.55	0.94	1.42	0.06	246,671	0.45	0.82	1.56	0.07	454,549
Diastolic blood pressure, automated reading (mmHg)	DBP	4079-0.0 - 4079-2.1 [$]	Age, age^2	145,363	42.00	83.98	145	9.81	188,110	32.00	80.03	138.5	9.77	333,473
Heel bone mineral density T-score, automated (standard deviation units)	hBMD	78-0.0	Age, age^2	119,681	-5.63	-0.08	8.42	1.33	142,107	-5.62	-0.59	5.81	1.03	261,788
Fluid intelligence score	FI	20016-0.0 - 20016-2.0	Age, age^2	76,360	0.00	6.31	13.00	2.17	89,084	0.00	6.07	13.00	2.02	165,444
Neuroticism score	Neuroticism	20127-0.0	Age, age^2	171,734	0.00	3.60	12.00	3.18	197,139	0.00	4.57	12.00	3.23	368,873
Qualifications (converted to years of education as in [27])	EA	6138-0.0 - 6138-2.5	YOB (as factor)	206,377	7.00	15.30	20.00	5.02	244,987	7.00	14.59	20.00	4.89	451,364
Skin colour	Skin	1717-0.0	-	203,556	1.00	2.15	4.00	0.55	244,086	1.00	2.12	4.00	0.56	447,642
Hair colour (natural, before greying)	Hair	1747-0.0	-	195,967	1.00	2.46	4.00	0.79	232,085	1.00	2.31	1.00	0.72	428,052
White blood cell (leukocyte) count (10^9 cells per litre)	WBC	30000-0.0 - 30000-2.0	Age, age^2	203,018	0.00	6.91	19.96	1.76	239,783	0.00	6.85	18.37	1.73	442,801
Platelet count (10^9 cells per litre)	Platelet	30080-0.0 - 30080-2.0	Age, age^2	203,042	2.4	237.49	573.50	53.88	239,793	0.4	265.40	624.90	58.91	442,835
Red blood cell (erythrocyte) count (10^{12} cells per litre)	RBC	30010-0.0 - 30010-2.0	Age, age^2	203,120	2.52	4.73	6.87	0.36	239,835	2.34	4.32	6.30	0.33	442,955
Haemoglobin concentration (grams per decilitre)	Hg	30020-0.0 - 30020-2.0	Age, age^2	203,129	8.90	15.00	20.52	1.00	239,840	7.82	13.52	19.20	0.93	442,969
Haematocrit percentage (%)	Hcrit	30030-0.0 - 30030-2.0	Age, age^2	203,122	25.5	43.30	60.7	2.95	239,832	22.7	39.28	55.72	2.73	442,954

^ WHR =Waist circumference [48-0.0 - 48-2.0] / Hip circumference [49-0.0 - 49-2.0]

[$] Individuals taking medications with blood pressure lowering effect were excluded from the analysis

*Age = Age of attending assessment centre [21003.0.0-21003.2.0], YOB = Year of birth [34-0.0]

[#]SD=standard deviation of the phenotype after adjusting for covariates, before scaling

Supplementary Table 1. B) The UK Biobank trait information. *N*, sample size

Trait	*N* male	*N* female
**Smoking**		
Cases (previous/current)	107,083	101,905
Controls (never)	100,453	144,250
**Skin colour**		
1=Very fair	13,678	23,065
2=Fair	149,805	171,329
3=Light olive	35,707	47,001
4=Dark olive	4,366	2,691
NA=Brown, Black, Do not know, Prefer not to answer		
**Hair colour (natural, before greying)**		
1=Blonde	20,374	30,978
2=Light brown	81,395	103,338
3=Dark brown	77,747	93,583
4=Black	16,451	4,186
NA=Red, Other, Do not know, Prefer not to answer		

Supplementary Table 2. The X-chromosome-wide (non-PAR) SNP-heritability (h^2_{SNP}) estimates and dosage compensation ratios (DCR) estimates obtained with GREML analysis and estimated from GWAS summary statistics.

**Trait**	**GREML *N***		**GREML**			**Summary statistics**
	**Male**	**Female**	$h^2_{SNP,M}$ **(SE, %)**	$h^2_{SNP,F}$ **(SE, %)**	$h^2_{SNP,M}/h^2_{SNP,F}$ **(SE)**	**DCR (SE)**
Height	99,762	99,796	1.55 (0.12)	0.88 (0.09)	1.76 (0.23)	1.59 (0.07)
FEV1	91,543	89,326	0.57 (0.10)	0.36 (0.09)	1.60 (0.47)	1.35 (0.16)
Smoking	99,566	99,584	0.48 (0.09)	0.18 (0.07)	2.67 (1.19)	1.98 (0.42)
Grip	99,651	99,551	0.42 (0.08)	0.28 (0.07)	1.52 (0.51)	1.78 (0.21)
BMI	99,634	99,663	0.97 (0.11)	0.42 (0.08)	2.28 (0.52)	2.13 (0.21)
Fat%	98,070	98,362	0.97 (0.11)	0.38 (0.08)	2.53 (0.62)	2.85 (0.32)
BMR	98,162	98,321	1.22 (0.12)	0.58 (0.09)	2.12 (0.40)	2.54 (0.19)
WHR	99,727	99,798	0.53 (0.09)	0.16 (0.07)	3.30 (1.48)	1.84 (0.25)
DBP	93,673	93,414	0.38 (0.09)	0.27 (0.08)	1.45 (0.55)	1.11 (0.26)
hBMD	90,779	90,800	0.52 (0.09)	0.27 (0.07)	1.95 (0.64)	2.13 (0.33)
FI	59,641	59,650	0.57 (0.13)	0.45 (0.12)	1.25 (0.45)	1.13 (0.27)
Neuroticism	98,925	95,683	0.38 (0.08)	0.17 (0.07)	2.19 (1.00)	1.51 (0.34)
EA	99,023	99,147	0.33 (0.08)	0.45 (0.09)	0.72 (0.22)	0.82 (0.13)
Skin	97,746	98,743	0.2 (0.07)	0.03 (0.06)	5.82 (9.46)	1.95 (0.74)
Hair	94,080	93,995	0.23 (0.07)	0.02 (0.05)	10.02 (23.45)	2.05 (0.76)
WBC	97,422	96,987	0.52 (0.09)	0.17 (0.07)	3.07 (1.36)	2.53 (0.46)
Platelet	97,426	96,991	0.55 (0.09)	0.19 (0.07)	2.90 (1.10)	2.55 (0.38)
RBC	97,460	97,009	0.61 (0.10)	0.26 (0.07)	2.36 (0.77)	2.46 (0.34)
Hgb	97,474	97,006	0.77 (0.11)	0.26 (0.08)	2.91 (0.92)	5.22 (0.77)
Hcrit	97,466	97,007	0.74 (0.11)	0.16 (0.07)	4.51 (2.00)	5.07 (0.83)
**Mean (SD)**	**95,361**	**95,042**	**0.63 (0.33)**	**0.30 (0.20)**	**2.85 (2.04)**	**2.23 (1.13)**

N, sample size; SE, standard error. **Traits:** standing height (Height), forced expiratory volume in 1-second (FEV1), smoking status (Smoking), hand grip strength, right (Grip), body mass index (BMI), body fat percentage (Fat%), basal metabolic rate (BMR), waist to hip ratio (WHR), diastolic blood pressure (DBP), heel bone mineral density T-score (hBMD), fluid intelligence score (FI), neuroticism score (Neuroticism), educational attainment (EA), skin colour (Skin), hair colour (Hair), white blood cell (leukocyte) count (WBC), platelet count (Platelet), red blood cell (erythrocyte) count (RBC), haemoglobin concentration (Hgb), Haematocrit percentage (Hcrit).

Supplementary Table 3. Estimated dosage compensation ratios (DCR) and genetic correlations (r_g) on the X chromosome and autosomes.

**Trait**	**X chromosome**		**Autosomes**	
	**DCR (SE)**	r_g **(SE)**	**DCR (SE)**	r_g **(SE)**
Height	1.59 (0.07)	0.96 (0.009)	0.98 (0.01)	0.96 (0.001)
FEV1	1.35 (0.16)	0.93 (0.030)	1.01 (0.02)	0.96 (0.003)
Smoking	1.98 (0.42)	0.95 (0.059)	1.04 (0.03)	0.85 (0.005)
Grip	1.78 (0.21)	0.82 (0.031)	1.17 (0.03)	0.86 (0.004)
BMI	2.13 (0.21)	0.80 (0.030)	1.02 (0.02)	0.94 (0.002)
Fat%	2.85 (0.32)	0.57 (0.053)	1.00 (0.02)	0.89 (0.002)
BMR	2.54 (0.19)	0.92 (0.018)	1.13 (0.01)	0.94 (0.002)
WHR	1.84 (0.25)	0.75 (0.039)	0.83 (0.02)	0.72 (0.004)
DBP	1.11 (0.26)	0.88 (0.067)	0.80 (0.02)	0.91 (0.005)
hBMD	2.13 (0.33)	0.97 (0.043)	0.66 (0.01)	0.91 (0.004)
FI	1.13 (0.27)	0.81 (0.069)	0.96 (0.03)	1.00 (0.006)
Neuroticism	1.51 (0.34)	0.94 (0.067)	0.94 (0.03)	0.90 (0.006)
EA	0.82 (0.13)	0.94 (0.040)	0.94 (0.02)	0.93 (0.004)
Skin	1.95 (0.74)	0.81 (0.120)	0.84 (0.02)	0.98 (0.003)
Hair	2.05 (0.76)	0.72 (0.129)	0.92 (0.01)	0.99 (0.002)
WBC	2.53 (0.46)	0.76 (0.053)	0.90 (0.01)	0.96 (0.003)
Platelet	2.55 (0.38)	0.78 (0.040)	0.91 (0.01)	0.96 (0.002)
RBC	2.46 (0.34)	0.64 (0.068)	0.94 (0.01)	0.93 (0.003)
Hgb	5.22 (0.77)	0.65 (0.044)	1.03 (0.02)	0.91 (0.004)
Hcrit	5.07 (0.83)	0.51 (0.050)	1.03 (0.02)	0.91 (0.004)
**Mean (SD)**	**2.23 (1.13)**	**0.81 (0.14)**	**0.95 (0.12)**	**0.92 (0.06)**

SE, standard error. **Traits:** standing height (Height), forced expiratory volume in 1-second (FEV1), smoking status (Smoking), hand grip strength, right (Grip), body mass index (BMI), body fat percentage (Fat%), basal metabolic rate (BMR), waist to hip ratio (WHR), diastolic blood pressure (DBP), heel bone mineral density T-score (hBMD), fluid intelligence score (FI), neuroticism score (Neuroticism), educational attainment (EA), skin colour (Skin), hair colour (Hair), white blood cell (leukocyte) count (WBC), platelet count (Platelet), red blood cell (erythrocyte) count (RBC), haemoglobin concentration (Hgb), Haematocrit percentage (Hcrit).

Supplementary Table 4. Regions of heterogeneity.

Region	Top SNP	Top-SNP bp	Heterogeneity *P*-value	Left Bound (bp)	Right Bound(bp)	Span (kb)	Trait
**Region 1**	rs17307280	8,916,646	4.34E-12	8,635,709	8,929,104	293	hBMD
**Region 1**	rs112265145	8,906,893	2.10E-44	8,635,709	8,929,104	293	Hgb
**Region 1**	rs56066690	8,912,070	8.45E-46	8,635,709	8,929,104	293	Hcrit
**Region 1**	rs56066690	8,912,070	1.83E-27	8,635,709	8,929,104	293	RBC
**Region 1**	rs745535498	8,912,871	6.62E-09	8,635,709	8,929,104	293	Fat%
**Region 2**	rs12556728	30,402,866	2.61E-08	30,320,507	30,572,217	251	Height
**Region 3**	rs56908677	56,958,534	4.97E-09	55,058,361	65,331,684	10,273	Hgb
**Region 3**	rs56908677	56,958,534	6.93E-09	55,058,361	65,331,684	10,273	Hcrit
**Region 4**	rs113121621	66,389,189	4.00E-08	56,197,395	67,837,267	11,639	Fat%

Traits: heel bone mineral density T-score (hBMD), haemoglobin concentration (Hgb), haematocrit percentage (Hcrit), red blood cell (erythrocyte) count (RBC), body fat percentage (Fat%), standing height (Height).

Supplementary Table 5. Estimates of dosage compensation ratios (DCR) and genetic correlation (r_g) after excluding the SNPs in the regions of heterogeneity.

Trait	DCR0	r_g 0	DCR1	r_g 1	DCR2	r_g 2	DCR3	r_g 3	DCR4	r_g 4	DCR13	r_g 13	DCR14	r_g 14
**hBMD**	2.13 (0.33)	0.97 (0.043)	2.08 (0.33)	0.98 (0.02)	-	-	-	-	-	-	-	-	-	-
**Fat%**	2.85 (0.32)	0.57 (0.053)	2.81 (0.32)	0.57 (0.03)	-	-	-	-	2.21 (0.26)	0.74 (0.02)	-	-	2.16 (0.26)	0.74 (0.02)
**Hgb**	5.22 (0.77)	0.65 (0.044)	4.87 (0.73)	0.68 (0.02)	-	-	2.54 (0.42)	0.68 (0.02)	-	-	2.15 (0.37)	0.74 (0.02)	-	-
**Hcrit**	5.07 (0.83)	0.51 (0.05)	4.66 (0.76)	0.53 (0.02)	-	-	2.53 (0.43)	0.62 (0.03)	-	-	2.12 (0.38)	0.68 (0.03)	-	-
**Height**	1.59 (0.07)	0.96 (0.009)	-	-	1.58 (0.07)	0.97 (0.003)	-	-	-	-	-	-	-	-
**RBC**	2.46 (0.34)	0.64 (0.068)	2.27 (0.32)	0.67 (0.04)	-	-	-	-	-	-	-	-	-	-

Numbers in parentheses indicate standard error. The DCR and r_g are marked as follows: 0 - including all SNPs, 1- excluding the SNPs in the region 1; 2- excluding the SNPs in the region 2; 3- excluding the SNPs in the region 3; 4- excluding the SNPs in the region 4; 13- excluding the SNPs in the region 1 and region 3; 14- excluding the SNPs in the region 1 and region 4. **Traits:** heel bone mineral density T-score (hBMD), body fat percentage (Fat%), haemoglobin concentration (Hgb), haematocrit percentage (Hcrit), standing height (Height), red blood cell (erythrocyte) count (RBC).

Supplementary Table 6. Number of lead SNPs identified in sex-stratified and combined analyses (GCTA-COJO [3]).

	Male discovery		Female discovery		Combined discovery	
	**Non-PAR**	**PAR**	**Non-PAR**	**PAR**	**Non-PAR**	**PAR**
BMI	10	--	2	--	19	1
BMR	23	--	4	--	37	1
DBP	0	--	0	--	1	--
EA	1	--	0	--	5	--
Fat%	10 (7)	--	2	--	16 (13)	1
FEV1	5	--	3	--	14	--
Grip	6	--	1	--	10	--
Hair	1	--	1	--	5	--
Hcrit	8 (6)	--	4	--	15 (13)	--
hBMD	4 (3)	--	2	--	5 (4)	1
Height	46 (45)	2	24	7	64 (63)	11
Hgb	6 (4)	--	3	--	13 (11)	--
Neuroticism	0	--	0	--	3	--
Platelet	13	--	8	--	15	--
RBC	9 (8)	--	3	1	16 (15)	1
Skin	1	--	0	--	1	--
Smoking	3	--	1	--	3	--
WBC	5	--	1	--	9	--
WHR	2	--	2	--	10	--
**Total**	**153 (143)**	**2**	**61**	**8**	**261 (251)**	**16**

The number of SNPs retained after exclusion of markers located in the regions of male-female heterogeneity for six traits is indicated parentheses. Non-PAR, non-pseudoautosomal region; PAR, pseudoautosomal region. **Traits:** standing height (Height), forced expiratory volume in 1-second (FEV1), smoking status (Smoking), hand grip strength, right (Grip), body mass index (BMI), body fat percentage (Fat%), basal metabolic rate (BMR), waist to hip ratio (WHR), diastolic blood pressure (DBP), heel bone mineral density T-score (hBMD), fluid intelligence score (FI), neuroticism score (Neuroticism), educational attainment (EA), skin colour (Skin), hair colour (Hair), white blood cell (leukocyte) count (WBC), platelet count (Platelet), red blood cell (erythrocyte) count (RBC), haemoglobin concentration (Hgb), Haematocrit percentage (Hcrit).

Supplementary Methods

Theoretical framework

Following [28], the genetic variance contributed by an X-chromosome SNP, under the assumption of Hardy-Weinberg equilibrium (HWE), in females is,

$$var(\beta_f X_f) = \beta_f^2 var(X_f) = 2p(1-p)\beta_f^2 \quad (1)$$

where, β_f is the per-allele effect estimate from a regression of SNP, X_f, on phenotype, y_f, with $X_f \in \{0,1,2\}$; and p, the minor allele frequency. Similarly, in males,

$$var(\beta_m X_m) = \beta_m^2 var(X_m) = p(1-p)\beta_m^2 \quad (2)$$

where, β_m is the per-allele effect estimate from a regression of SNP, X_m, on phenotype, y_m, with $X_m \in \{0,1\}$. Dosage compensation can be parameterised as $\beta_m = d\beta_f$, where $1 \leq d \leq 2$. In general,

$$var(\beta_m X_m) = \beta_m^2 var(X_m) = p(1-p)\beta_m^2 = d^2 p(1-p)\beta_f^2 \quad (3)$$

Under a full dosage compensation model ($d = 2$), $\beta_m = 2\beta_f$ and,

$$var(\beta_m X_m) = \beta_m^2 var(X_m) = p(1-p)\beta_m^2 = 4p(1-p)\beta_f^2 \quad (4)$$

That is, the variance contributed by a X-linked SNP in males is twice that of females. Under a no dosage compensation model ($d = 1$), $\beta_m = \beta_f$ and,

$$var(\beta_m X_m) = \beta_m^2 var(X_m) = p(1-p)\beta_m^2 = p(1-p)\beta_f^2 \quad (5)$$

That is, the variance contributed by a X-linked SNP in males is half that of females. Further, we can estimate d (i.e. dosage compensation ratio) by exploiting the following relationship,

$$E[\chi_i^2] = 1 + \frac{N_i h_i^2}{M_{eff}} \quad (6)$$

for $i \in \{m, f\}$, where, $E[\chi_i^2]$ is the expected mean χ_i^2 statistic; N_i is the sample size; h_i^2 is the proportion of variance explained by X-chromosome SNPs; and M_{eff} is the effective number of X-chromosome SNPs. Rearranging for h_i^2 and taking the ratio $\hat{\gamma} = \frac{h_m^2}{h_f^2}$, we get,

$$\hat{\gamma} = \frac{h_m^2}{h_f^2} = \frac{(\hat{\chi}_m^2 - 1)N_f}{(\hat{\chi}_f^2 - 1)N_m} \quad (7)$$

where $\hat{\gamma}$ ranges between 0.5 (i.e. no dosage compensation) and 2 (i.e. full dosage compensation). Finally, the expectation of the cross-product of the z-statistics from the male and female analyses, χ^2_{mf} is,

$$E[\chi^2_{mf}] = \frac{r_g h_m h_f N_M N_f}{M_{eff}} \quad (8)$$

where r_g is the genetic correlation between males and females. Rearranging,

$$E[\chi^2_i] = 1 + \frac{N_i h^2_i}{M_{eff}} \quad (9)$$

for h^2_i and substituting, we get,

$$\hat{r}_g = \frac{\hat{\chi}^2_{mf}}{\sqrt{(\hat{\chi}^2_m - 1)(\hat{\chi}^2_f - 1)}} \quad (10)$$

UK Biobank Data

Sample selection. The complex trait analysis was conducted utilizing the UK Biobank (UKB) data (available to researchers upon application; see URLs). We inferred ancestries of 488,377 genotyped participants of the UKB as described in [29], and a dataset of European-ancestry individuals that met our sample quality inclusion criteria (*N*=455,605) was taken forward for the analysis. The samples were excluded according to UKB provided information if: (i) the genetically inferred sex was inconsistent with the submitted gender, (ii) there was evidence for putative sex chromosome aneuploidy, (iii) samples were reported as heterozygosity and missingness outliers, (iv) were excluded from kinship inference, or if participants have withdrawn their consent for use of their the data.

Genotype data. The imputed genotypes for both autosomes and X-chromosome pseudo-autosomal (PAR, coded as chromosome 25) and non-PAR (coded as chromosome 23) regions are available as a part of the UKB Version 3 release of the genotype data. Individuals were genotyped on either Affymetrix UK BiLEVE Axiom (*N*=50,000) or the Affymetrix UK Biobank Axiom® array (*N*=450,000). The genotypes were imputed to UK10K+1000GP3 and HRC reference panels and include both SNPs and small indels [30]. We further hard-called the provided genotype probabilities (chromosomes 1-22, 23 and 25) of non-multiallelic markers with info-score >0.3, treating the calls with uncertainty >0.1 as missing, and keeping the markers which meet our quality control criteria in the set of unrelated European individuals (HWE test $P<10^{-6}$ and missing call rate <5%). The heterozygous calls in non-PAR region of the X chromosome male genotypes were set to missing. To avoid deflation of heritability estimates on the X chromosome, we only analyse the markers with minor allele frequency (MAF) >0.01 in our full sample of European participants. We estimates allele frequencies (AF) of the X-chromosome markers for both sexes and retained the common set of 6,871 PAR and

253,842 non-PAR SNPs. Similarly, for the comparison of the X-chromosomal and autosomal results for height and BMI, we conducted autosomal association analyses with a two-fold reduced sample size and using 8,546,066 markers on chromosomes 1-22 with MAF>0.01.

Phenotype selection. A total of 20 complex traits were selected for analysis in the UKB. All analyses, as well as phenotype adjustment, were performed on a sex-specific basis. The phenotypes were adjusted for covariates and the residuals were transformed to sex-specific Z-scores (mean=0, variance=1) with the measured phenotypic values over 6 standard deviations (SD) away from the mean previously removed from the analysis. For individuals with repeated measures of the phenotype, we estimated the mean value of the observed measures after outlier removal procedure for each assessment visit, and used mean age across the visits as a covariate. For each trait, the UK Biobank variable identifiers, available sample sizes and covariates are presented in Supplementary Table 1A, as well as the minimum, maximum and mean values of the raw phenotype measures and the standard deviations of the phenotype after adjustment for trait-specific covariates. The discrete phenotypes (educational attainment, smoking status, skin and hair colours) were treated as quantitative (see Supplementary Table 1B for description of the categories) in our association analysis.

Consortium for the Architecture of Gene Expression (CAGE) data

Gene expression and X-chromosome genotype data. Gene expression and X-chromosome genotype data were available in a subset of N=2,130 individuals (N=1,084 males, N=1,046 females) from the Consortium for the Architecture of Gene Expression (CAGE), a study examining the genetic architecture of gene expression in a mixture of pedigree and unrelated individuals [31]. This subset of individuals comes from three cohorts with genotype data on the X chromosome [32–35], and are of European ancestry, as identified by principal component analysis with the HapMap3 populations. Further details are provided in [31].

Quality control of gene expression data. RNA was collected from whole blood samples in each cohort and gene expression levels quantified using the Illumina Whole-Genome Expression BeadChips (HT12 v.3 and HT12 v.4). A total of 38,624 gene expression probes were common to all cohorts. Gene expression quality control and normalisation was performed in each cohort separately before concatenation. This included variance stabilisation and quantile normalisation to standardise the distribution of expression levels across samples. To remove hidden and known experimental confounders, gene expression levels were then adjusted for a mean of 39/50 probabilistic estimation of expression residuals (PEER) factors [36,37] across the three cohorts that were not associated with sex (P_{sex_diff}>0.05) in order to preserve the effect of sex on expression, and where available, measured covariates such as age, cell counts, and batch effects. Residuals for each cohort were then standardised to Z-scores and concatenated across cohorts. The concatenated gene expression dataset was further adjusted for 18/50 PEER factors that were not associated with sex (P_{sex_diff}>0.05) and standardised to Z-scores. A total of 36,267 autosomal and 1,639 X-chromosome gene expression probes

(corresponding to 26,384 and 1,138 unique genes, respectively) that unambiguously mapped to the genome formed our final gene expression dataset. This included a total of 28 PAR X-chromosome gene expression probes.

Quality control and imputation of genotype data. Genotype data was acquired using different genotyping platforms for each cohort, with quality control performed within each cohort before concatenation. Details for autosomal quality control and imputation are provided in [31]. Briefly, autosomal SNPs were imputed to the 1000 Genomes Phase 1 Version 3 reference panel [38] within each cohort and concatenated resulting in 7,763,174 SNPs passing quality control, which included filtering SNPs for MAF<0.01, HWE test $P<10^{-6}$, and imputation info score <0.3. This set of imputed autosomal SNPs was further filtered to 1,066,905 HapMap3 SNPs that were common to all three cohorts. This set of imputed autosomal SNPs formed our final dataset. For each cohort, we used the Sanger Imputation Server (see URLs) to impute SNPs on the non-PAR of the X chromosome to the Haplotype Reference Consortium (HRC, release 1.1) [39], using the EAGLE2+PBWT pre-phasing and imputation pipeline [40,41]. Pre-imputation checks included ensuring all alleles are on the forward strand, and coordinates and reference alleles are on the GRCh37 assembly. Pre-imputation quality control included filtering X-chromosome genotyped SNPs for MAF<0.01, HWE test $P<10^{-6}$ within females, SNP missingness call rate >2%, and genotyped SNPs that are not in the HRC reference panel. A total of 1,228,034 X-chromosome SNPs were available following imputation in each cohort. Post-imputation quality control within cohort included filtering imputed X-chromosome SNPs for MAF<0.01, HWE test $P<10^{-6}$ within females, imputation info score <0.3, and multiallelic SNPs. A total of 306,589 imputed X-chromosome SNPs were common to all cohorts and formed the concatenated dataset. We performed further quality control of the concatenated dataset by filtering imputed X-chromosome SNPs for missingness call rate >2%. A total of 190,506 imputed X-chromosome SNPs remained. Additional post-imputation quality control on the concatenated dataset included a comparison of allele frequencies between males and females, which led to the exclusion of 261 SNPs with MAF differences of >0.05 between sexes. A total of 190,245 imputed X-chromosome SNPs formed our final dataset.

Genotype Tissue Expression (GTEx) data

We used the Genotype Tissue Expression project (GTEx v6p release) dataset, comprised of RNA-seq data from 39 non-diseased tissue-types for which a sex covariate was available in N=449 deceased human donors, as an external validation of our X-chromosome *cis*-eQTL results across multiple tissue-types. The fully-processed, normalised and filtered RNA-seq GTEx v6p data were downloaded from the GTEx Portal (https://www.gtexportal.org/home/datasets) along with corresponding covariate files. X-chromosome imputed SNP data was obtained from dbGap (Accession phs000424.v6.p1). Briefly, gene expression normalisation included filtering for transcripts with at least 10 samples with RPKM >0.1 and raw read counts greater than 6, quantile normalisation within tissue, and inverse quantile normalisation for each transcript. Sample outliers were identified and excluded

using a correlation-based statistic described in [42], and samples with less than 10 million mapped reads were excluded. Further details can be found in [43]. Quality control of the X-chromosome imputed SNP data included filtering for MAF<0.05, HWE test $P<10^{-6}$ within females, imputation info score <0.4, and multiallelic SNPs. A total of 127,808 imputed SNPs in the non-PAR of the X chromosome were included in our analysis. We restricted our analyses to 22 tissue samples for which within tissue sample size was greater than N=50 in both males and females (Supplementary data 6). Sample sizes per tissue ranged from N=124 in colon (sigmoid) to N=361 in muscle (skeletal) with a mean of N=226 across the 22 tissues. The proportion of males and females within each tissue ranged from 34% females in heart (atrial appendage) to 44% females in adrenal gland, with a mean of 38% females across all 22 tissues. A total of 1,121 X-linked transcripts (including 31 PAR transcripts) were expressed in at least one of the 22 tissues. The number of X-linked transcripts identified as expressed in each tissue ranged from 726 in pancreas to 916 in thyroid, with a mean of 808 across all 22 tissues (Supplementary data 6).

Statistical Analysis

GWAS. To determine the dosage compensation (DC) ratios across 20 complex traits and to compare effect sizes of genome-wide significant X-chromosome markers on those phenotypes, we analyse the results of X-chromosome wide analysis (XWAS) (both PAR and non-PAR) performed on a sex-specific basis using BOLT-LMM v2.3 [44] in the full set of UKB European males (N_m=208,419) and females (N_f =247,186). We include a set of HapMap3 SNPs (MAF>0.01 and pairwise R^2<0.9 in the window of 1000 SNPs) in the mixed model to correct for the population stratification and to account for relatedness. This set of model SNPs (M=561,572) includes autosomal markers, 12,508 non-PAR and 205 PAR SNPs on the X chromosome. All other X-chromosome SNPs are fixed effects and tested for association using linear regression. For the comparison of the X-chromosomal and autosomal results for height and BMI, the autosomal association analyses were performed in a sample of ~104,000 males (N= 103,983 for height, N=103847 for BMI). The above described set of 548,860 autosomal HapMap3 markers were used as model SNPs and 7,997,206 additional non-GRM SNPs were included for association testing.

Combined analyses. The choice of the optimum meta- and combined male-female analyses depends on the assumptions of dosage compensation and the genotype coding in males [28]. While the true extent of dosage compensation is not known, its effect can be parameterised as $\beta_m = d\beta_f$, with d being a dosage compensation parameter ($d = 1$ for no dosage and $d = 2$ for full dosage compensation). In the sex-stratified analysis, we regress a phenotype on a genotype variable, where $X_f \in \{0,1,2\}$ for females and $X_m \in \{0, c\}$ in males, with $c = 1$ in the no DC analysis or $c = 2$ in the full DC analysis (i.e. assuming full random X-inactivation). When $c = 1$, we estimate per-allele effects in males. From the Eq. 4.6 and 4.7 in [28], it follows that an optimum meta-analysis of the estimates from the sex-stratified analysis is only unbiased when $d = c$. That is, under a no DC model, the meta- and combined male-female analyses will

be unbiased when using per-allele effect estimates in males ($c = 1$), while under a full DC model, they are unbiased when the effect estimates in males are from an association analysis where the male genotypes coded as diploid ($c = 2$). Since the results from our sex-stratified analysis are largely consistent with expectations from full dosage compensation, we perform an inverse variance weighted meta-analysis for complex traits using the male effect size estimates from the diploid analysis to obtain the joint estimates of the SNP effects, and in the combined analyses of gene expression traits we code males as diploids.

Sex differences in gene expression. Sex differences in gene expression were examined with a mixed linear regression model implemented in the GCTA software package [25]. Here, we tested for sex differences in gene expression for 1,639 X-linked gene expression probes. Gene expression was modelled as,

$$\mathbf{y} = \boldsymbol{\mu} + \mathbf{X}\beta + \mathbf{g_G} + \mathbf{g_X} + \boldsymbol{\varepsilon} \tag{11}$$

where $\mathbf{y}$ is a N x 1 vector of gene expression intensity levels; $\boldsymbol{\mu}$ is the mean expression levels; β is the regression coefficient for the fixed sex covariate, $\mathbf{X}$, with males coded as 1 and females coded as 2; $\mathbf{g_G}$ is an N x 1 vector of the total genetic effects of the individuals with $\mathbf{g_G} \sim N(0, \mathbf{A_G}\sigma_G^2)$, where $\mathbf{A_G}$ is interpreted as the autosomal GRM between individuals calculated from 1,066,905 HapMap3 SNPs; $\mathbf{g_X}$ is an N x 1 vector of X-linked genetic effects with $\mathbf{g_X} \sim N(0, \mathbf{A_X}\sigma_X^2)$, where $\mathbf{A_X}$ is a GRM calculated from 190,506 imputed X-chromosome SNPs; and $\boldsymbol{\varepsilon} \sim N(0, \mathbf{I}\sigma_\epsilon^2)$ is the residual. We used the Wald statistic to assess significance, and calculated a P-value by comparing the test statistic to a χ^2-distribution with one degree of freedom.

X-chromosome *cis*-eQTL analysis. To investigate the X-chromosome genetic control of gene expression, we modelled gene expression levels as a linear function of the number of reference alleles in a linear mixed regression model, in males and females separately and in a combined male-female analysis, using the GCTA software package [25]. The model for each gene expression probe can be written as,

$$\mathbf{y} = \boldsymbol{\mu} + \mathbf{X}\boldsymbol{\beta} + \mathbf{g_G} + \boldsymbol{\varepsilon} \tag{12}$$

where, $\mathbf{y}$ is a N x 1 vector of gene expression intensity levels, with sample size N; $\boldsymbol{\beta}$ is a vector of fixed effect estimates for the indicator variable for the genotype, $\mathbf{X}$; $\mathbf{g_G}$ is an N x 1 vector of the total genetic effects of the individuals with $\mathbf{g_G} \sim N(0, \mathbf{A_G}\sigma_G^2)$, where $\mathbf{A_G}$ is interpreted as the autosomal genetic relationship matrix (GRM) between individuals calculated from the 1,066,905 HapMap3 SNPs; and $\boldsymbol{\varepsilon} \sim N(0, \mathbf{I}\sigma_\epsilon^2)$ is the residual. Since our interest is in testing for the association between X-chromosome SNPs and gene expression, this is equivalent to a leave-one-chromosome-out analysis [45]. To assess significance, we calculated a Wald test statistic and calculated a P-value (P_{eQTL}) by comparing the test statistic to a χ^2-distribution with one degree of freedom. We accounted for multiple testing for both the number of X-chromosome SNPs and the number of gene expression probes tested using the Bonferroni method. For each gene expression probe, eQTLs were defined as the top associated X-chromosome SNP that satisfies the Bonferroni significance threshold of $P_{\text{eQTL}}<1.6\text{x}10^{-10}$ (i.e.

0.05/(1,639x190,245) in the discovery sex. The XCI status (Escape/Variable or Inactive) for the identified eQTLs were assigned by mapping gene expression probes to XCI status using the gene name from [26].

Autosomal *cis*-eQTL analysis. We compared results from our sex stratified X-chromosome *cis*-eQTL analysis to the autosome by performing an autosomal *cis*-eQTL analysis in males and females, separately. Here, we model autosomal gene expression levels as a linear function of the number of reference alleles for autosomal SNPs on the same chromosome using the GCTA software package [25]. Each autosomal gene expression probe is modelled in the same way as described in Eq. 12. We identified eQTLs as probe-SNP pairs with $P_{\text{eQTL}}<10^{-10}$ in the discovery sex.

X-chromosome *cis*-eQTL analysis in GTEx. We modelled gene expression as a linear function of the number of reference alleles in a linear regression model for males and females separately using PLINK [46]. The model for each X-chromosome transcript can be written as,

$$\mathbf{y} = \boldsymbol{\mu} + \mathbf{X}\boldsymbol{\beta} + \boldsymbol{\varepsilon} \quad (13)$$

where, $\mathbf{y}$ is a N x 1 vector of gene expression intensity levels, with sample size N; $\boldsymbol{\beta}$ is a vector of fixed effect estimates for the for the indicator variable for the genotype, $\mathbf{X}$; and $\boldsymbol{\varepsilon}\sim N(0, \mathbf{I}\sigma_{\epsilon}^{2})$ is the residual. The model was adjusted for three genotyping principal components (PCs) and PEER factors, which captures batch effects and latent experimental confounders in the gene expression data. Following [43], a total of 15 PEER factors were included in the model for total sample sizes N<150, 30 PEER factors for total sample sizes 150≤N<250, and 35 PEER factors for total sample sizes N≥250. To assess significance, we calculated a t-statistic and calculated a P-value by comparing the test statistic to the t-distribution. We identified eQTLs as transcript-SNP pairs that satisfied the within tissue Bonferroni significance threshold, which accounts for both the number of X-linked transcripts and X-chromosome SNPs tested in each tissue in the discovery sex (Supplementary data 6). DCC was estimated in each of the 22 tissue-types as described in the main methods. The XCI status (Escape/Variable or Inactive) for the identified eQTLs in each tissue was assigned by mapping transcript gene identifiers from [26]. We tested for enrichment of Escape/Variable status in each tissue using a hypergeometric test. As the proportion of males and females within each tissue is highly skewed towards males, sensitivity analysis included randomly removing male samples from the analysis so that the proportions match that of females within each of the tissues. This is repeated 100 times, with DCC calculated across the 100 replicates. We also identified the top eQTLs among all tissues in the discovery sex, and extracted the corresponding eQTL from the same tissue in the other sex. DCC is calculated as described in the main methods.

Finally, we performed a 2 degree-of-freedom eQTL interaction analysis with males and females combined. We modelled gene expression as a linear function of SNP, sex, and SNP-by-sex interaction, adjusting for genotyping PCs and PEER factors (as described above). To

assess significance, we compared the interaction model to a null model without the SNP and SNP-by-sex interaction effects using the *anova* function in R. Similar to our main analysis, we identified eQTLs as transcript-SNP pairs that satisfied the within tissue Bonferroni significance threshold. The regression coefficients for these transcript-SNP pairs were then extracted in the male- and female-only analyses, and DCC was estimated in each of the 22 tissue-types as described in the main methods.

URLs

GTEx, see https://www.gtexportal.org/home/
UK Biobank, see http://www.ukbiobank.ac.uk/
Sanger Imputation Server, see https://imputation.sanger.ac.uk/

References

1. Yang, J. *et al*. Genome partitioning of genetic variation for complex traits using common SNPs. *Nat. Genet.* **43,** 519–525 (2011).
2. Lee, J. J. *et al*. Gene discovery and polygenic prediction from a genome-wide association study of educational attainment in 1.1 million individuals. *Nat. Genet.* **50,** 1112–1121 (2018).
3. Yang, J. *et al*. Conditional and joint multiple-SNP analysis of GWAS summary statistics identifies additional variants influencing complex traits. *Nat. Genet.* **44,** 369 (2012).
4. Ohlsson, C. *et al*. Genetic determinants of serum testosterone concentrations in men. *PLoS Genet.* **7,** (2011).
5. Pirastu, N. *et al*. GWAS for male-pattern baldness identifies 71 susceptibility loci explaining 38% of the risk. *Nat. Commun.* **8,** (2017).
6. Pickrell, J. K. *et al*. Detection and interpretation of shared genetic influences on 42 human traits. *Nat. Genet.* **48,** 709–717 (2016).
7. Martinez-Garay, I. *et al*. A new gene family (FAM9) of low-copy repeats in Xp22.3 expressed exclusively in testis: Implications for recombinations in this region. *Genomics* **80,** 259–267 (2002).
8. Shahani, S., Braga-Basaria, M., Maggio, M. & Basaria, S. Androgens and erythropoiesis: Past and present. *Journal of Endocrinological Investigation* **32,** 704–716 (2009).
9. De Pergola, G. The adipose tissue metabolism: role of testosterone and dehydroepiandrosterone. *Int. J. Obes. Relat. Metab. Disord.* **24 Suppl 2,** S59–S63 (2000).
10. Clarke, B. L. & Khosla, S. Androgens and bone. *Steroids* **74,** 296–305 (2009).
11. Jadhav, U., Harris, R. M. & Jameson, J. L. Hypogonadotropic hypogonadism in subjects with DAX1 mutations. *Molecular and Cellular Endocrinology* **346,** 65–73 (2011).
12. Dangle, P. *et al*. Female-to-male sex reversal associated with unique Xp21.2 deletion disrupting genomic regulatory architecture of the dosage-sensitive sex reversal region. *J. Med. Genet.* **54,** 705–709 (2017).
13. Smyk, M. *et al*. Male-to-female sex reversal associated with an ~250 kb deletion upstream of NR0B1 (DAX1). *Hum. Genet.* **122,** 63–70 (2007).
14. Bardoni, B. *et al*. A dosage sensitive locus at chromosome Xp21 is involved in male to female sex reversal. *Nat. Genet.* **7,** 497–501 (1994).
15. Evans, D. M. *et al*. Genome-wide association study identifies loci affecting blood copper, selenium and zinc. *Hum. Mol. Genet.* **22,** 3998–4006 (2013).
16. Prasad, A. S., Mantzoros, C. S., Beck, F. W. J., Hess, J. W. & Brewer, G. J. Zinc status and serum testosterone levels of healthy adults. *Nutrition* **12,** 344–348 (1996).
17. Houghton, L. A., Parnell, W. R., Thomson, C. D., Green, T. J. & Gibson, R. S. Serum Zinc Is a Major Predictor of Anemia and Mediates the Effect of Selenium on

Hemoglobin in School-Aged Children in a Nationally Representative Survey in New Zealand. *J. Nutr.* **146,** 1670–1676 (2016).

18. Jin, H. J., Kim, J. & Yu, J. Androgen receptor genomic regulation. *Transl Androl Urol* (2013). doi:10.3978/j.issn.2223-4683.2013.09.01

19. Wei, H. *et al.* Sumoylation delimits KLF8 transcriptional activity associated with the cell cycle regulation. *J. Biol. Chem.* (2006). doi:10.1074/jbc.M513135200

20. Liu, Y.-N. *et al.* KLF4 functions as an activator of the androgen receptor through reciprocal feedback. *Oncogenesis* (2016). doi:10.1038/oncsis.2016.79

21. Eaton, S. A. *et al.* A network of Krüppel-like factors (Klfs): Klf8 is repressed by Klf3 and activated by Klf1 in vivo. *J. Biol. Chem.* (2008). doi:10.1074/jbc.M804831200

22. Van Hout, C. V *et al.* Whole exome sequencing and characterization of coding variation in 49,960 individuals in the UK Biobank. *bioRxiv* 572347 (2019). doi:10.1101/572347

23. Tukiainen, T. *et al.* Chromosome X-Wide Association Study Identifies Loci for Fasting Insulin and Height and Evidence for Incomplete Dosage Compensation. *PLoS Genet.* **10,** (2014).

24. Zhu, Z. *et al.* Integration of summary data from GWAS and eQTL studies predicts complex trait gene targets. *Nat. Genet.* **48,** 481–487 (2016).

25. Yang, J., Lee, S. H., Goddard, M. E. & Visscher, P. M. GCTA: A tool for genome-wide complex trait analysis. *Am. J. Hum. Genet.* **88,** 76–82 (2011).

26. Tukiainen, T. *et al.* Landscape of X chromosome inactivation across human tissues. *Nature* **550,** 244–248 (2017).

27. Okbay, A. *et al.* Genome-wide association study identifies 74 loci associated with educational attainment. *Nature* **533,** 539–542 (2016).

28. Lee, J. J. *et al.* Gene discovery and polygenic prediction from a genome-wide association study of educational attainment in 1.1 million individuals. *Nature Genetics* (2018). doi:10.1038/s41588-018-0147-3

29. Yengo, L. *et al.* Meta-analysis of genome-wide association studies for height and body mass index in ~700000 individuals of European ancestry. *Hum. Mol. Genet.* **27,** 3641–3649 (2018).

30. Bycroft, C. *et al.* The UK Biobank resource with deep phenotyping and genomic data. *Nature* (2018). doi:10.1038/s41586-018-0579-z

31. Lloyd-Jones, L. R. *et al.* The Genetic Architecture of Gene Expression in Peripheral Blood. *Am. J. Hum. Genet.* **100,** 228–237 (2017).

32. Kim, J. *et al.* Gene expression profiles associated with acute myocardial infarction and risk of cardiovascular death. *Genome Med.* **6,** 40 (2014).

33. Leitsalu, L. *et al.* Cohort profile: Estonian biobank of the Estonian genome center, university of Tartu. *Int. J. Epidemiol.* **44,** 1137–1147 (2015).

34. Powell, J. E. *et al.* Congruence of Additive and Non-Additive Effects on Gene Expression Estimated from Pedigree and SNP Data. *PLoS Genet.* **9,** 1–10 (2013).

35. Powell, J. E. *et al.* The Brisbane systems genetics study: Genetical genomics meets complex trait genetics. *PLoS One* **7,** (2012).

36. Stegle, O., Parts, L., Piipari, M., Winn, J. & Durbin, R. Using probabilistic estimation of expression residuals (PEER) to obtain increased power and interpretability of gene expression analyses. *Nat. Protoc.* **7,** 500–507 (2012).

37. Stegle, O., Parts, L., Durbin, R. & Winn, J. A bayesian framework to account for complex non-genetic factors in gene expression levels greatly increases power in eQTL studies. *PLoS Comput. Biol.* **6,** 1–11 (2010).

38. Altshuler, D. M. *et al.* An integrated map of genetic variation from 1,092 human genomes. *Nature* **491,** 56–65 (2012).

39. McCarthy, S. *et al.* A reference panel of 64,976 haplotypes for genotype imputation. *Nat. Genet.* **48,** 1279–1283 (2016).

40. Loh, P. R. *et al.* Reference-based phasing using the Haplotype Reference Consortium panel. *Nat. Genet.* **48,** 1443–1448 (2016).

41. Durbin, R. Efficient haplotype matching and storage using the positional Burrows-Wheeler transform (PBWT). *Bioinformatics* **30,** 1266–1272 (2014).

42. Wright, F. A. *et al.* Heritability and genomics of gene expression in peripheral blood. *Nat. Genet.* **46,** 430–437 (2014).

43. Consortium, Gte. Genetic effects on gene expression across human tissues. *Nature* **550,** 204–213 (2017).

44. Loh, P. R., Kichaev, G., Gazal, S., Schoech, A. P. & Price, A. L. Mixed-model association for biobank-scale datasets. *Nature Genetics* **50,** 906–908 (2018).

45. Yang, J., Zaitlen, N. A., Goddard, M. E., Visscher, P. M. & Price, A. L. perspective Advantages and pitfalls in the application of mixed-model association methods. *Nat. Publ. Gr.* **46,** 100–106 (2014).

46. Purcell, S. *et al.* PLINK: A Tool Set for Whole-Genome Association and Population-Based Linkage Analyses. *Am. J. Hum. Genet.* **81,** 559–575 (2007).
