# Supplementary material for: The effect of X-linked dosage compensation on complex trait variation
Source: Nat Commun. 2019 Jul 8;10:3009. doi: 10.1038/s41467-019-10598-y (PMC6614401; doi:10.1038/s41467-019-10598-y)
Supplement: Supplementary file 2 — Description of Additional Supplementary Files [file 41467_2019_10598_MOESM2_ESM.docx]

**Description of Supplementary Files**

**File Name:** **Supplementary Data 1**

**Description:** Lead SNPs from the male-specific discovery analysis in the UK Biobank. Summary statistics for the lead SNPs (selected in conditional and joint analysis, GCTA-COJO, P_COJO_<5x10^-8^) for 20 complex traits from the UK Biobank male-specific XWAS analysis. Shown are the chromosome number (CHR, 23 refers to the non-pseudoautosomal region on the X chromosome and 25 to the pseudoautosomal region); trait; SNP; base-pair position (BP); effect allele (refA), frequency of the effect allele in the discovery sex (freq); estimated per-allele effect size (b_male) corresponding standard error (se_male) and association *P*-value (P_male) from the sex-specific XWAS analysis in males; estimated per-allele effect size (b_fem), corresponding standard error (se_fem) and association *P*-value (P_fem) from the sex-specific XWAS analysis in females; heterogeneity *P*-value (PHet) for the markers within regions of heterogeneity for the 6 traits with significant male-female heterogeneity. Physical gene (Gene.phys) for the markers that are physically located within a gene region; X-chromosome inactivation status (XCI.status) of the physical gene; ratio of the male-female SNP effect size estimates (Effect size ratio) and the corresponding standard error (SE).

**File Name: Supplementary Data 2**

**Description:** Lead SNPs from the female-specific discovery analysis in the UK Biobank. Summary statistics for the lead SNPs (selected in conditional and joint analysis, GCTA-COJO, P_COJO_<5x10^-8^) for 20 complex traits from the UK Biobank female-specific XWAS analysis. Shown are the chromosome number (CHR, 23 refers to the non-pseudoautosomal region on the X chromosome and 25 to the pseudoautosomal region); trait; SNP; base-pair position (BP); effect allele (refA), frequency of the effect allele in the discovery sex (freq); estimated per-allele effect size (b_male) corresponding standard error (se_male) and association *P*-value (P_male) from the sex-specific XWAS analysis in males; estimated per-allele effect size (b_fem), corresponding standard error (se_fem) and association *P*-value (P_fem) from the sex-specific XWAS analysis in females; heterogeneity *P*-value (PHet) for the markers within regions of heterogeneity for the 6 traits with significant male-female heterogeneity. Physical gene (Gene.phys) for the markers that are physically located within a gene region; X-chromosome inactivation status (XCI.status) of the physical gene; ratio of the male-female SNP effect size estimates (Effect size ratio) and the corresponding standard error (SE).

**File Name:** **Supplementary Data 3**

**Description:** Lead SNPs from the combined male-female discovery analysis in the UK Biobank. Summary statistics for the lead SNPs (selected in conditional and joint analysis, GCTA-COJO, P_COJO_<5x10^-8^) for 20 complex traits from the combined male-female meta-analysis (under full dosage compensation model) in the UK Biobank. Shown are the chromosome number (CHR, 23 refers to the non-pseudoautosomal region on the X chromosome and 25 to the pseudoautosomal region); trait; SNP; base-pair position (BP); effect allele (refA), mean male-female frequency of the effect allele weighted by sample size (freq); estimated combined effect size (b_comb) with corresponding standard error (se_comb) and association *P*-value (P_comb) form the male-female meta-analysis; estimated per-allele effect size (b_male) corresponding standard error (se_male) and association *P*-value (P_male) from the sex-specific XWAS analysis in males; estimated per-allele effect size (b_fem), corresponding standard error (se_fem) and association *P*-value (P_fem) from the sex-specific XWAS analysis in females; heterogeneity *P*-value (PHet) for the markers within regions of heterogeneity for the 6 traits with significant male-female heterogeneity. Physical gene (Gene.phys) for the markers that are physically located within a gene region; X-chromosome inactivation status (XCI.status) of the physical gene; ratio of the male-female SNP effect size estimates (Effect size ratio) and the corresponding standard error (SE).

**File Name:** **Supplementary Data 4**

**Description:** CAGE, male discovery eQTL analysis. Summary statistics for top associated SNPs for each of the 1,639 X-linked gene expression probes from the CAGE male-specific analysis. Shown are the probe and SNP positions; frequency of the effect allele (A1) in males (Freq.mal) and females (Freq.fem); the per-allele effect estimate (b.mal) and corresponding standard error (se.mal) and p-value (p.mal) in males; the per-allele effect estimate (b.fem) and corresponding standard error (se.fem) and p-value (p.fem) in females; and the inactivation status of the gene (XCI.status).

**File Name:** **Supplementary Data 5**

**Description:** CAGE, female discovery eQTL analysis. Summary statistics for top associated SNPs for each of the 1,639 X-linked gene expression probes from the CAGE female-specific analysis. Shown are the probe and SNP positions; frequency of the effect allele (A1) in males (Freq.mal) and females (Freq.fem); the per-allele effect estimate (b.mal) and corresponding standard error (se.mal) and p-value (p.mal) in males; the per-allele effect estimate (b.fem) and corresponding standard error (se.fem) and p-value (p.fem) in females; and the inactivation status of the gene (XCI.status).

**File Name:** **Supplementary Data 6**

**Description:** Samples size and number of X-linked transcripts expressed per tissue-type in GTEx. X-chromosome *cis*-eQTL analysis is performed in 22 tissue samples for which within tissue sample size was greater than *N*=50 in both males and females. Shown are the total sample sizes in each tissue (Total); the total number of females (No. Females) and males (No. males); and the number of X-linked transcripts (No. X-linked transcripts) included in the analysis.

**File Name:** **Supplementary Data 7**

**Description:** eQTL analysis across tissues in GTEx. Estimated dosage compensation coefficients (DCC) and corresponding standard errors (SE) for 22 tissues in GTEx in the male and female discovery analysis. Shown are the number of eQTLs (No. eQTLs) that were used to calculate DCC; the number of eQTLs annotated as escape/variable genes (No. Esc/Var eQTLs); the number of eQTLs annotated as inactive genes (No. Inac. eQTLs); and *P*-value from an enrichment test for escape/variable transcript-SNP pairs from a hypergeometric test. The male discovery analysis was down-sampled to match the sample size that of females, with the mean DCC, the standard deviation (SD), and mean number of eQTLs calculated across 100 replicates.

**File Name:** **Supplementary Data 8**

**Description:** eQTL interaction analysis across tissues in GTEx. eQTLs (No. eQTLs) were identified by a 2 degree-of-freedom combined male-female interaction analysis across 22 tissues in GTEx. The regression coefficients for these eQTLs were extracted in the male- and female-only analyses, and the dosage compensation coefficient (DCC) and the corresponding standard error (SE) were estimated.

**File Name:** **Supplementary Data 9**

**Description:** Results from the combined male-female summary-data based Mendelian randomisation (SMR) analysis. Summary statistics for the SNPs identified in the SMR analysis on the X chromosome (P_SMR_<3x10^-5^ and P_HEIDI_>0.05). SMR prioritizes genes whose expression levels are associated with complex traits due to pleiotropy for 20 complex traits in the UK Biobank and eQTL data from CAGE whole blood (combined male-female analyses, under full dosage compensation model). Shown are the trait, probe ID (probeID), chromosome (ProbeChr), base pair position of the probe (Probe_bp) and corresponding gene; top SNP name, base pair position, the effect allele (A1) and other allele (A2), frequency of the effect allele (estimated from the reference sample); effect size (b_GWAS), standard error (se_GWAS) and *P*-value (p_GWAS) from GWAS; effect size (b_eQTL), standard error (se_eQTL) and *P*-value (p_GWAS) from eQTL study; effect size (b_SMR), standard error (se_SMR) and *P*-value (p_SMR) from SMR, *P*-value from HEIDI (HEterogeneity In Dependent Instruments) test (p_HEIDI), and number of SNPs used in the HEIDI test (nsnp_HEIDI); estimated per-allele effect size (b_male) and corresponding standard error (se_male) from the sex-specific XWAS analysis in males; estimated per-allele effect size (b_fem) and corresponding standard error (se_fem) from the sex-specific XWAS analysis in females; reported inactivation status (XCI.status) for the SMR gene; Physical gene (Gene.phys) for the markers that are physically located within a gene region; reported inactivation status (XCI.status) for the physical gene; ratio of the male-female SNP effect size estimates (Effect size ratio) and the corresponding standard error (SE).

**File Name:** **Supplementary Data 10**

**Description:** Results from the male-specific summary-data based Mendelian randomisation (SMR) Analysis. Summary statistics for the SNPs identified in the SMR analysis on the X chromosome (P_SMR_<3x10^-5^ and P_HEIDI_>0.05). SMR prioritizes genes whose expression levels are associated with complex traits due to pleiotropy for 20 complex traits in the UK Biobank and eQTL data from CAGE whole blood (male-specific analyses). Shown are the trait, probe ID (probeID), chromosome (ProbeChr), base pair position (Probe_bp) and corresponding gene; top SNP name, base pair position, the effect allele (A1) and other allele (A2), frequency of the effect allele (estimated from the reference sample); effect size (b_GWAS), standard error (se_GWAS) and *P*-value (p_GWAS) from GWAS; effect size (b_eQTL), standard error (se_eQTL) and *P*-value (p_GWAS) from eQTL study; effect size (b_SMR), standard error (se_SMR) and *P*-value (p_SMR) from SMR, *P*-value from HEIDI (HEterogeneity In Dependent Instruments) test (p_HEIDI), and number of SNPs used in the HEIDI test (nsnp_HEIDI); estimated per-allele effect size (b_male) and corresponding standard error (se_male) from the sex-specific XWAS analysis in males; estimated per-allele effect size (b_fem) and corresponding standard error (se_fem) from the sex-specific XWAS analysis in females; reported inactivation status (XCI.status) for the SMR gene; Physical gene (Gene.phys) for the markers that are physically located within a gene region; reported inactivation status (XCI.status) for the physical gene; ratio of the male-female SNP effect size estimates (Effect size ratio) and the corresponding standard error (SE).

**File Name:** **Supplementary Data 11**

**Description:** Results from the female-specific summary-data based Mendelian randomisation (SMR) analysis. Summary statistics for the SNPs identified in the SMR analysis on the X chromosome (P_SMR_<3x10^-5^ and P_HEIDI_>0.05). SMR prioritizes genes whose expression levels are associated with complex traits due to pleiotropy for 20 complex traits in the UK Biobank and eQTL data from CAGE whole blood (female-specific analyses). Shown are the trait, probe ID (probeID), chromosome (ProbeChr), base pair position (Probe_bp) and corresponding gene; top SNP name, base pair position, the effect allele (A1) and other allele (A2), frequency of the effect allele (estimated from the reference sample); effect size (b_GWAS), standard error (se_GWAS) and *P*-value (p_GWAS) from GWAS; effect size (b_eQTL), standard error (se_eQTL) and *P*-value (p_GWAS) from eQTL study; effect size (b_SMR), standard error (se_SMR) and *P*-value (p_SMR) from SMR, *P*-value from HEIDI (HEterogeneity In Dependent Instruments) test (p_HEIDI), and number of SNPs used in the HEIDI test (nsnp_HEIDI); estimated per-allele effect size (b_male) and corresponding standard error (se_male) from the sex-specific XWAS analysis in males; estimated per-allele effect size (b_fem) and corresponding standard error (se_fem) from the sex-specific XWAS analysis in females; reported inactivation status (XCI.status) for the SMR gene; Physical gene (Gene.phys) for the markers that are physically located within a gene region; reported inactivation status (XCI.status) for the physical gene; ratio of the male-female SNP effect size estimates (Effect size ratio) and the corresponding standard error (SE).

**File Name:** **Supplementary Data 12**

**Description:** Results from the combined male-female summary-data based Mendelian randomisation (SMR) analysis for the *ITM2A* gene. *ITM2A* gene-trait association results (P_SMR_<3x10^-5^ and not filtered on P_HEIDI_). SMR prioritizes genes whose expression levels are associated with complex traits due to pleiotropy for 20 complex traits in the UK Biobank and eQTL data from CAGE whole blood (combined male-female analyses, under full dosage compensation model). Shown are the trait, probe ID (probeID), chromosome (ProbeChr), base pair position (Probe_bp) and corresponding gene; top SNP name, base pair position, the effect allele (A1) and other allele (A2), frequency of the effect allele (estimated from the reference sample); effect size (b_GWAS), standard error (se_GWAS) and *P*-value (p_GWAS) from GWAS; effect size (b_eQTL), standard error (se_eQTL) and *P*-value (p_GWAS) from eQTL study; effect size (b_SMR), standard error (se_SMR) and *P*-value (p_SMR) from SMR, *P*-value from HEIDI (Heterogeneity In Dependent Instruments) test (p_HEIDI), and number of SNPs used in the HEIDI test (nsnp_HEIDI); estimated per-allele effect size (b_male) and corresponding standard error (se_male) from the sex-specific XWAS analysis in males; estimated per-allele effect size (b_fem) and corresponding standard error (se_fem) from the sex-specific XWAS analysis in females; reported inactivation status (XCI.status) for the SMR gene; Physical gene (Gene.phys) for the markers that are physically located within a gene region; reported inactivation status (XCI.status) for the physical gene; ratio of the male-female SNP effect size estimates (Effect size ratio) and the corresponding standard error (SE).

**File Name:** **Supplementary Data 13**

**Description:** Results from the combined male-female summary-data based Mendelian randomisation (SMR) analysis in *trans* regions on the X chromosome. Summary statistics for the SNPs identified in the SMR analysis on the X chromosome (P_SMR_<3x10^-5^ and P_HEIDI_>0.05). SMR prioritizes genes whose expression levels are associated with complex traits due to pleiotropy for 20 complex traits in the UK Biobank and eQTL data from CAGE whole blood (combined male-female analyses, under full dosage compensation model). Shown are the trait, gene, probe ID (probeID), chromosome (ProbeChr) and base pair position (Probe_bp); *trans*-eQTL chromosome (trans_chr), left boundary of the *trans*-region (trans_leftBound), right boundary of the *trans*-region (trans_rightBound), top SNP name, base pair position, the effect allele (A1) and other allele (A2), frequency of the effect allele (estimated from the reference sample); effect size (b_GWAS), standard error (se_GWAS) and *P*-value (p_GWAS) from GWAS; effect size (b_eQTL), standard error (se_eQTL) and *P*-value (p_GWAS) from eQTL study; effect size (b_SMR), standard error (se_SMR) and *P*-value (p_SMR) from SMR, *P*-value from HEIDI (Heterogeneity In Dependent Instruments) test (p_HEIDI), and number of SNPs used in the HEIDI test (nsnp_HEIDI); estimated per-allele effect size (b_male) and corresponding standard error (se_male) from the sex-specific XWAS analysis in males; estimated per-allele effect size (b_fem) and corresponding standard error (se_fem) from the sex-specific XWAS analysis in females; reported inactivation status (XCI.status) for the SMR gene; Physical gene (Gene.phys) for the markers that are physically located within a gene region; reported inactivation status (XCI.status) for the physical gene; ratio of the male-female SNP effect size estimates (Effect size ratio) and the corresponding standard error (SE).
